# A System-Wide Investigation and Stratification of the Hemostatic Proteome in Premature Myocardial Infarction

**DOI:** 10.3389/fcvm.2022.919394

**Published:** 2022-06-30

**Authors:** Joanne L. Dunster, Joy R. Wright, Nilesh J. Samani, Alison H. Goodall

**Affiliations:** ^1^School of Biological Sciences, Institute for Cardiovascular and Metabolic Research, Reading, United Kingdom; ^2^Department of Cardiovascular Sciences, University of Leicester & NIHR Leicester Biomedical Research Centre, Glenfield Hospital, Leicester, United Kingdom

**Keywords:** computational biology, coagulation, thrombosis, myocardial infarction, clinical studies, gender

## Abstract

**Introduction:**

Advancing understanding of key factors that determine the magnitude of the hemostatic response may facilitate the identification of individuals at risk of generating an occlusive thrombus as a result of an atherothrombotic event such as an acute Myocardial Infarction (MI). While fibrinogen levels are a recognized risk factor for MI, the association of thrombotic risk with other coagulation proteins is inconsistent. This is likely due to the complex balance of pro- and anticoagulant factors in any individual.

**Methods:**

We compared measured levels of pro- and anticoagulant proteins in plasma from 162 patients who suffered an MI at an early age (MI <50 y) and 186 age- and gender-matched healthy controls with no history of CAD. We then used the measurements from these individuals as inputs for an established mathematical model to investigate how small variations in hemostatic factors affect the overall amplitude of the hemostatic response and to identify differential key drivers of the hemostatic response in male and female patients and controls.

**Results:**

Plasma from the MI patients contained significantly higher levels of Tissue Factor (*P* = 0.007), the components of the tenase (FIX and FVIII; *P* < 0.0001 for both) and the prothrombinase complexes (FX; *P* = 0.003), and lower levels of Tissue Factor Pathway Inhibitor (TFPI; *P* = 0.033) than controls. The mathematical model, which generates time-dependent predictions describing the depletion, activation, and interaction of the main procoagulant factors and inhibitors, identified different patterns of hemostatic response between MI patients and controls, and additionally, between males and females. Whereas, in males, TF, FVIII, FIX, and the inhibitor TFPI contribute to the differences seen between case and controls, and in females, FII, FVIII, and FIX had the greatest influence on the generation of thrombin. We additionally show that further donor stratification may be possible according to the predicted donor response to anticoagulant therapy.

**Conclusions:**

We suggest that modeling could be of value in enhancing our prediction of risk of premature MI, recurrent risk, and therapeutic efficacy.

## Introduction

Acute myocardial infarction (MI) is initiated by the rupture or erosion of an atherosclerotic plaque in a coronary artery, resulting in arterial thrombosis. This is driven by the combined interaction of platelets and plasma coagulation factors, resulting in thrombin generation and subsequent conversion of fibrinogen to fibrin to form a platelet-rich thrombus. While platelet reactivity (1, 2) and plasma levels of fibrinogen are recognized risk factors for MI (3–6), the overall extent and rate of coagulation may also be a key factor in determining the likelihood of generating an occlusive thrombus. The amplitude of the hemostatic response may have particular relevance in patients who suffer an MI at an early age when the atherosclerotic burden is relatively low.

Within the normal population, levels of coagulation factors vary several-fold between individuals. Some of this variation has been linked to Single Nucleotide Polymorphisms (SNPs); in particular, the non-synonymous SNP in the prothrombin gene (G20210G>A), which has been linked to elevated plasma levels of prothrombin (7) and increased risk of venous (7, 8) and arterial (9–11) thrombosis; and the Factor V Leiden mutation (F5 R506Q) that prevents activation of anticoagulant Protein C and thereby increases the risk of venous thrombosis (12–15). However, none of the SNPs in hemostatic proteins have emerged as risk alleles for coronary artery disease (CAD) or MI in the largest genome-wide studies (16, 17).

Previous studies associating plasma levels of individual components of the coagulation cascade with the risk of MI have been inconsistent. The independent association of Factor VII (FVII) with coronary heart disease reported in the Northwick Park Heart Study (3) was not replicated in either the PROCAM (4) or ARIC (18) studies. More robust evidence exists for an association between elevated levels of Factor VIII (FVIII) and coronary risk or recurrent MI (19–22); however, the gender difference in plasma levels of FVIII, which are elevated in females, is at odds with the lower risk of MI in females (21). This observation suggests that the dynamics that maintain the hemostatic balance may differ between males and females.

The interactions between individual pro-coagulation factors and inhibitors are complex, forming a network of positive and negative feedback that changes over time. Mathematical representations of the coagulation cascade have sought to produce predictions of these time-dependent variations (23–25). These models have predominantly used single values for each coagulation factor based on mean plasma levels in healthy individuals. Studies that model coagulation in cohorts of donors with clinical conditions are few (26–28). In addition, the majority of previous studies have used a fixed value for Tissue Factor (TF), the initiator of coagulation, predetermined from the plasma of healthy individuals. Since circulating levels of TF also vary and are known to be elevated in premature MI (pMI) patients (29), existing modeling may not truly represent the early stages of activation of coagulation.

We therefore measured endogenous levels of TF activity, as well as plasma levels of Factors II, V, VII, VIII, IX, and X and activities of two key inhibitors, Tissue Factor Pathway Inhibitor (TFPI) and Antithrombin (AT), in the plasma of 162 patients who suffered an MI under 50 years and 186 healthy subjects matched for age, gender, and smoking status, with no history of cardiovascular disease (30). We then analyzed the data in a previously validated mathematical model of coagulation (23) to generate computer predictions of the hemostatic response for each individual. The predictions highlight how each factor is activated, inhibited, and depleted, and also their influence on the hemostatic response over time. The generation of multi-dimensional data predicting the hemostatic response in a large cohort of pMI patients and healthy donors is novel and allows the identification of the differential key drivers of the hemostatic response in both populations and between males and females. The model also allowed stratification of donor response to anticoagulants, suggesting that further development of the model could be of value in enhancing the prediction of risk of pMI, recurrent risk, and potential therapeutic efficacy.

## Methods

### Study Cohorts

The pMI cases and controls were part of the PRAMIS cohort (30), reported previously. All had suffered a MI according to WHO criteria before the age of 50 years. At the time of participation, all case subjects were at least 6 months from their acute event and in a clinically stable condition. The control cohort had no history of cardiovascular disease and was matched for age, sex, and current smoking status with the cases. Controls were recruited from the same geographical area as the cases, and all subjects were Caucasian of Northern European origin. The study was approved by the Leicestershire Health Authority Ethics Committee and all subjects provided written informed consent. Plasma samples suitable for analysis for this study were available from 162 of the cases and 186 controls. Demographic and clinical data are provided in Table 1.

**Table 1:** Demographic variables in the PRAMIS cases and controls.

**Variable**	**Cases (*n* = 162)**	**Controls (*n* = 186)**	***P*-value**
Age (years)	47.5 ± 5.7	47.6 ± 5.7	0.8507
Male:Female (%)	86: 14	87: 13	0.9060
Event age (years)	42.7 ± 5.7	N/A	-
Current Smokers (%)	21	18	0.7864
Body Mass Index (kg/m^2^)	30.0 ± 8.1	27.0 ± 4.0	<0.0001
BP systolic (mm Hg)	130 ± 17.4	133 ± 11.6	0.0773
BP diastolic (mm Hg)	83 ± 11.2	86 ± 9.9	0.0076
Exercise (%) (none/1-2/>3 times /week)	45:43:12	32:42:26	0.0022
Hypertension (%)	28.4	9.7	<0.0001
Diabetes mellitus (%)	12.3	1.1	<0.0001
Dyslipidaemia (%)	87.7	9.7	<0.0001
Fibrinogen (mg/dL)	3.13 ± 0.76	2.83 ± 0.61	<0.0001
Total cholesterol (mmol/L)	5.15 ± 1.24	5.21 ± 1.05	0.6319
Triglycerides (mmol/L)	2.04 ± 0.98	1.52 ± 0.86	<0.0001
HDL cholesterol (mmol/L)	1.15 ± 0.34	1.36 ± 0.35	<0.0001
LDL cholesterol (mmol/L)	3.09 ± 1.00	3.16 ± 0.92	0.5016
CRP (mmol/L)	2.19 ± 3.74	2.04 ± 2.26	0.6354
Homocysteine (mmol/L)	12.61 ± 4.27	11.96 ± 4.14	0.1498
Lp(a) (mg/l)	147 ± 232	184 ± 225	0.0120

*BP, Blood Pressure; HDL, High Density Lipoprotein; LDL, Low Density Lipoprotein; CRP, C-Reactive Protein; Lp(a), Lipoprotein (a)*.

### Blood Collection

Fasting blood samples were collected by clean venepuncture via a 21-g butterfly needle without a tourniquet, into 3.2% (w/v) tri-sodium citrate and centrifuged within 10 min of collection at 1800 g for 30 min. Plasma was stored in single-use aliquots, at −80°C until analysis.

### Analysis of Plasma Clotting Factors

Plasma levels of coagulation factors II, V, VII, VIII, IX, and X were measured by one-stage turbidimetric clotting assays using a Sysmex CA6000 coagulation analyzer (Sysmex, Milton Keynes, UK) and factor-specific deficient plasmas from Dade Behring (Milton Keynes, UK). Clotting time was determined against a standard curve for each factor, and data were expressed as the percent of a reference plasma that was analyzed in parallel. Antithrombin (AT) was measured on the Sysmex CA6000 analyzer, using an automated chromogenic method (Berichrome; Dade Behring). Plasma TF and TFPI activity were measured using chromogenic assays based on the generation of FXa (ADI, Stamford, CT, USA). These were calculated as pM and units/ml, respectively, based on standards provided with the assays.

Genotyping of the prothrombin 20210G>A SNP was carried out in DNA samples from the PRAMIS cohort as described previously (31).

### Computational Methods

The mathematical model used to generate computational predictions has been previously published, validated, and used to investigate donor data (23, 26, 32, 33). For completeness, full details of the mathematical model (including the effect of anticoagulant drugs) are provided in the Supplementary Section SI. We also provide the code (details below) to run the models in R and MATLAB and, to aid researchers unfamiliar with running such code, have developed an online app to demonstrate how changes in levels of individual coagulation factors may affect the amplitude of the coagulation response (https://cardiomaths.shinyapps.io/ThrombinGeneration/).

**Figure 1:**
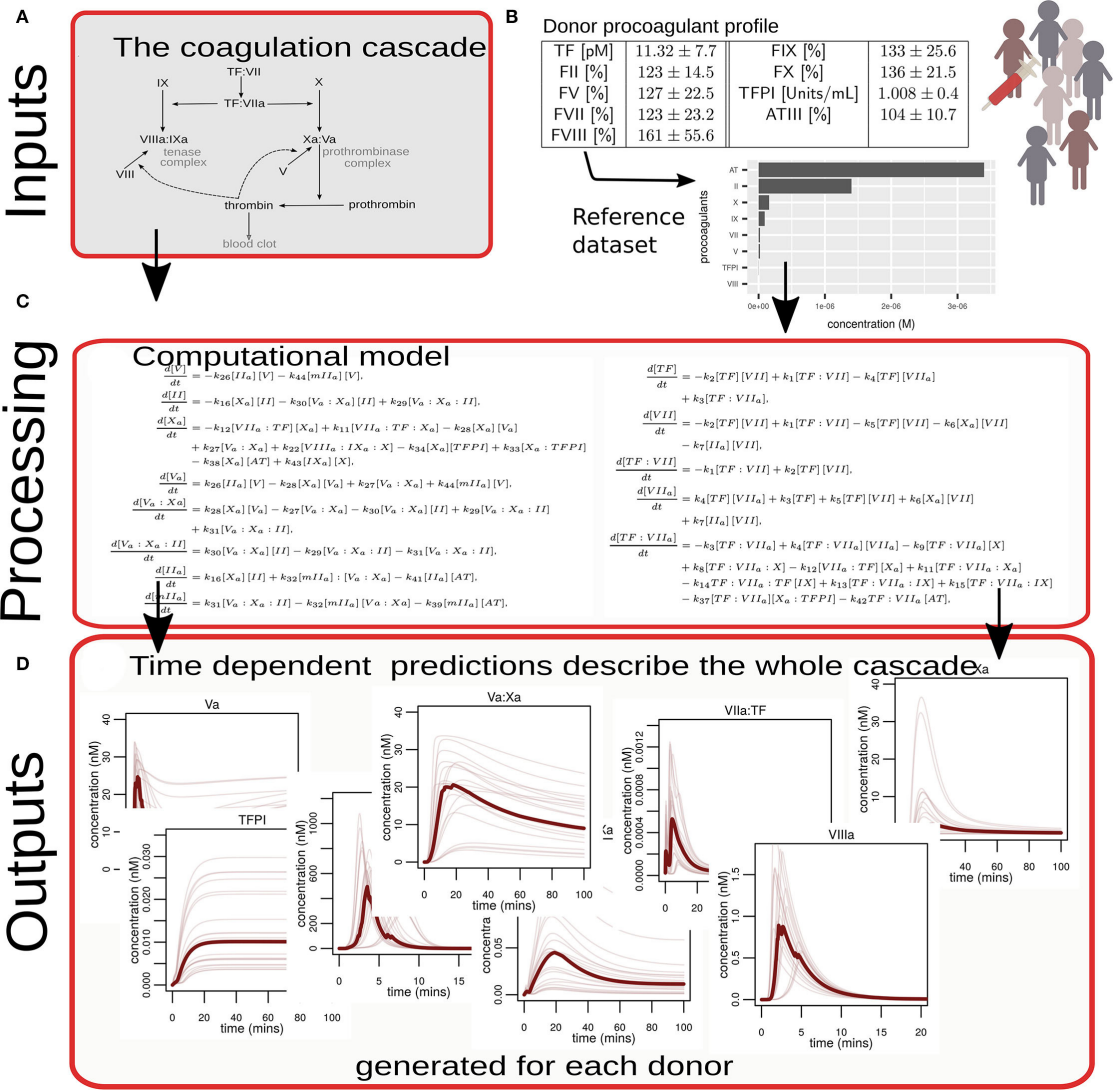
Diagrammatic overview of the process used to convert experimental data into computational simulations of coagulation. **(A)** A biological description (in the form of a network diagram) of the reactions that comprise the coagulation cascade that was translated into a mathematical model. **(B)** Illustrates that coagulation proteins in plasma from 348 individual donors were measured, converted via a reference dataset to absolute concentrations, and used as model inputs. **(C)** Examples of numerical simulations of the mathematical model provide predictions. The equations shown here are for illustrative purposes only. The complete set of equations is given in the Supplemental File. **(D)** Illustrates examples of how all the proteins and complexes that comprise the coagulation cascade change and interact over time. These predictions were generated for each donor.

The process used to generate predictions for this study, summarized in Figure 1, was as follows:

A. The reactions that comprise the coagulation cascade (described fully in the Supplemental File and illustrated schematically in Supplementary Figure SI) are converted under the assumption of mass action into a set of 34 ordinary differential equations, where each equation captures the changes for one species. For example, for the generation of FIIa by the prothrombinase (FVa:FXa) complex:

$$\frac{d\left[II\right]}{dt}=-k_{16}\left[Xa\right]\left[II\right]-k_{30}\left[Va:Xa\right]\left[II\right]+k_{29}\left[Va:Xa:II\right].$$

Here, *k*_16_ and *k*_30_ denote the rate by which prothrombin is converted to its activated form via the action of activated FX and by the prothrombinase complex, respectively, and *k*_29_ denotes the release of prothrombin from the prothrombinase complex.

B. Measured levels of coagulation factor activity from the 162 pMI patients and 186 healthy controls were used as inputs for the model. Each plasma coagulation factor value, measured as a percentage of a normal pooled control, was first converted into absolute concentrations using a reference dataset (Supplementary Table SI) (23).
C. Numerical solutions of the mathematical model, using known rates of reaction for each of the coagulation factors (Table SII) (23, 34, 35), were generated for each donor.
D. A set of 19 time-dependent summary predictions (Supplementary Section SII) were collected for each donor that describes the system-wide coagulation response covering the generation and depletion of all procoagulant proteins and inhibitors and their conversion into activated factors and complexes.

A heatmap to visualize the relative levels of procoagulant proteins and inhibitors measured for each donor was generated using the R package, complexHeatmap. In this, each row represents an individual donor and the donors have been divided into tertiles based on their predicted ability to generate thrombin.

### Statistical Analysis

Characteristics of cases and controls are shown as mean±SD unless otherwise stated. Data were compared using an unpaired *t*-test for continuous variables and the chi-squared test for categorical variables.

### Data and Software Availability

The code (R and MATLAB) to generate simulations from the mathematical model utilized in this paper is available via https://github.com/cardiomaths/coagSim and a version archived at the time of publication at https://doi.org/10.6084/m9.figshare.13007645.v1. An online app to run the model is available via https://cardiomaths.shinyapps.io/ThrombinGeneration/.

## Results

### Characteristics of the PRAMIS Subjects

The mean age at the time of recruitment was 47.5 ± 5.7 years for pMI cases and 47.6 ± 5.7 years for controls (*P* = 0.85) (Table 1). The mean age of patients at the time of MI was 42.7 ± 5.7 years (range 29–45 years). The cases were predominantly male (86%), and therefore the control cohort reflected this bias (87% male; *P* = 0.90). Many of the factors associated with the risk of MI were significantly different between the two groups, including body mass index (BMI) (higher in the cases; *P* ≤ 0.001), frequency of exercise (lower in cases; *P* = 0.002), plasma fibrinogen (higher in cases), and smoking history (higher in cases; *P* < 0.001). However, at the time of recruitment, the two groups were matched for smoking status with only 21% of cases and 18% of controls being current smokers (*P* = 0.39). All cases were taking aspirin (75 mg pd) but none was on any other antiplatelet drugs or anticoagulants. None of the controls were taking antiplatelet or anticoagulant medication. None of the cases or controls had a known bleeding disorder or thrombophilia.

### Plasma Coagulation Factors Levels

**Figure 2:**
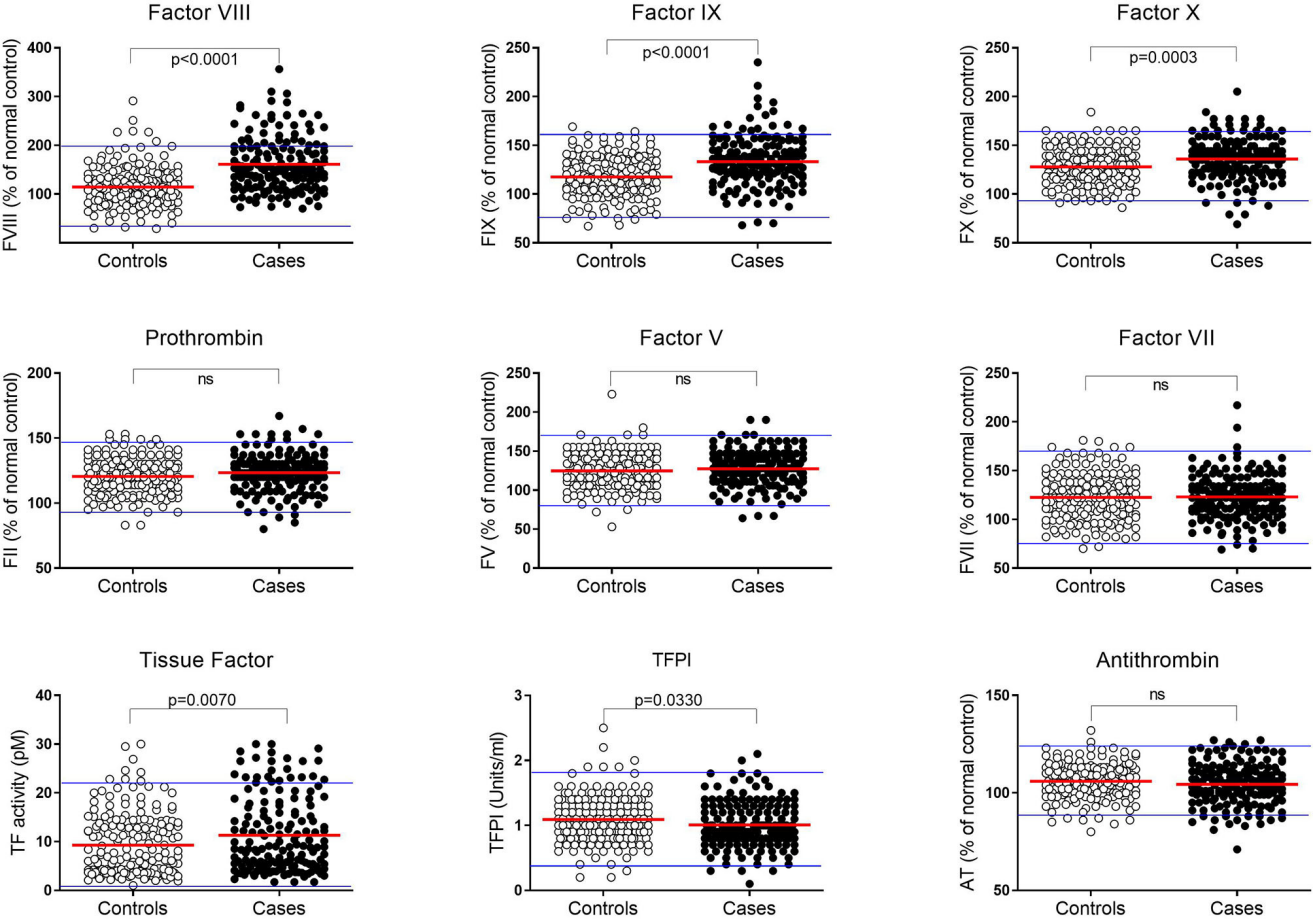
Plasma coagulation factor levels in premature MI subjects and controls. Levels of each coagulation factor were measured in plasma from pMI cases (*n* = 162; closed circles) and controls (*n* = 186; open circles). Factors II, V, VII, VIII, IX, X, and antithrombin (AT) are reported as the percentage of reference control plasma included in each analysis. Tissue Factor (TF) and Tissue Factor Pathway Inhibitor (TFPI) are reported as activity (pM) and (U/mL), respectively. A normal range for each protein is depicted by the upper and lower blue lines and is based on the mean value ± 2SDs in plasma from the healthy controls. (*P*-values derived by unpaired t-test after confirming data were normally distributed).

The levels of procoagulant FVIII, FIX, FX, FII, FV, FVII, and TF, and inhibitors TFPI and AT, measured in the plasma of all subjects are shown in Figure 2 and Supplementary Table SI. A normal range for each factor, representing the mean ± 2SD, was established based on data from the control group and is shown in Figure 2 by the blue horizontal lines. While the plasma levels of each clotting factor in the pMI cohort were predominantly within the normal range, procoagulant FVIII, FIX, FX, and TF were, on average, significantly higher (*P* ≤ 0.007 for all). In contrast, plasma levels of the inhibitor TFPI were significantly lower in the cases (*P* = 0.033). Levels of prothrombin (FII), FVII, FV, and antithrombin were not significantly different between the cases and controls.

### Simulations of Coagulation Profiles for Premature MI and Control Groups

**Figure 3:**
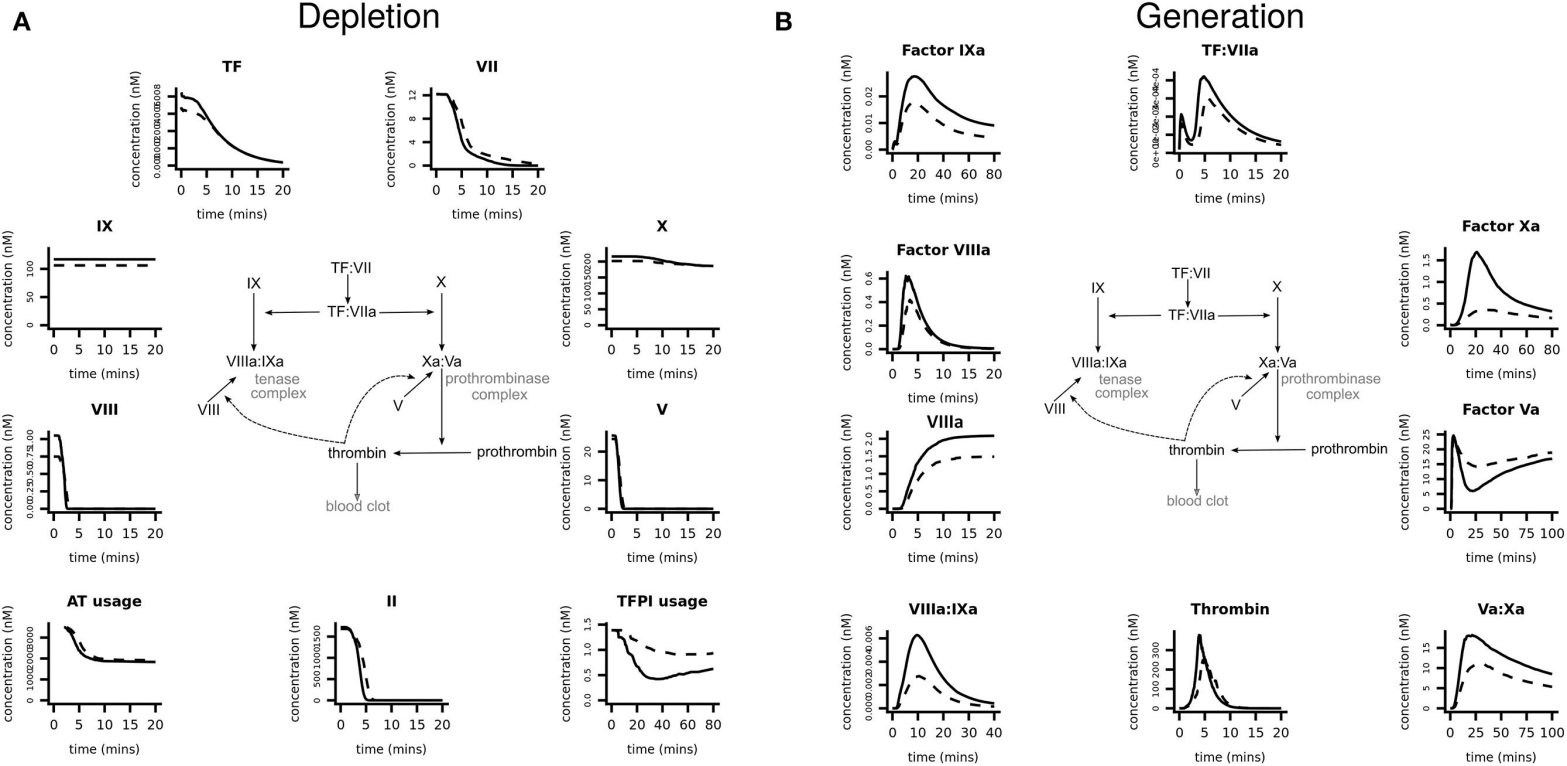
Prediction of coagulation protein depletion and generation in premature MI subjects vs. healthy donors. Computer simulations were generated, using the plasma protein values as inputs, to predict **(A)** the depletion and **(B)** the generation of coagulation proteins. Each graph shows the median simulated response predicted, specific to each coagulation protein or complex, for the populations of pMI subjects (solid lines) and healthy donors (broken lines). These data are provided in more detail in Supplementary Figures SII, SIII which show the median and 5% and 95% confidence intervals.

Coagulation factor levels were first converted into molar concentrations and these values were then used as inputs into the mathematical model to generate the system-wide, coagulation response for each pMI subject and healthy control. The simulations gave dynamic, time-dependent predictions of coagulation factor depletion (Figure 3A), and the generation of, and conversion into, activated factors and complexes (Figure 3B). For clarity, only the median response of each group (pMI–solid lines; control donors–broken lines) is shown; however, the variations in the responses in both groups are provided in the Supplementary Section (**Figures SII and SIII**). Supplementary Figure SIII also provides quantitative data on the difference between the cases and controls in the amount and time for the depletion of each factor.

The simulations in Figure 3A demonstrated that within 5 min, procoagulant factors V and VIII were rapidly depleted, followed by prothrombin, while FVII and TF took longer to become depleted (~15 and 20 min, respectively). FX and FIX were in excess and therefore not rate limiting. Whereas, pMI cases consumed, on average, 34% more TFPI than controls, the greatest difference between cases and controls was in the generation of activated coagulation factors (Figure 3B). While the median value for the generation of thrombin from prothrombin was 51% higher in cases than in controls, other activated factors provided a better discriminator between the two groups; for example, the generation of activated FIX (FIXa) was 57% higher, and of FXa was 376% higher in the MI donor cases. Interestingly, despite the availability of FXa, the limited availability of FV, and consequently of FVa, the cofactor of FXa in the prothrombinase complex would act as a brake on the cascade, restricting assembly of the prothrombinase complex and therefore limiting the downstream generation of thrombin. Therefore, the large differences in predicted FXa generation between cases and controls have little impact on the eventual thrombotic end point.

### A Single Procoagulant Factor Is Not Predictive of the Amplitude of the Hemostatic Response

**Figure 4:**
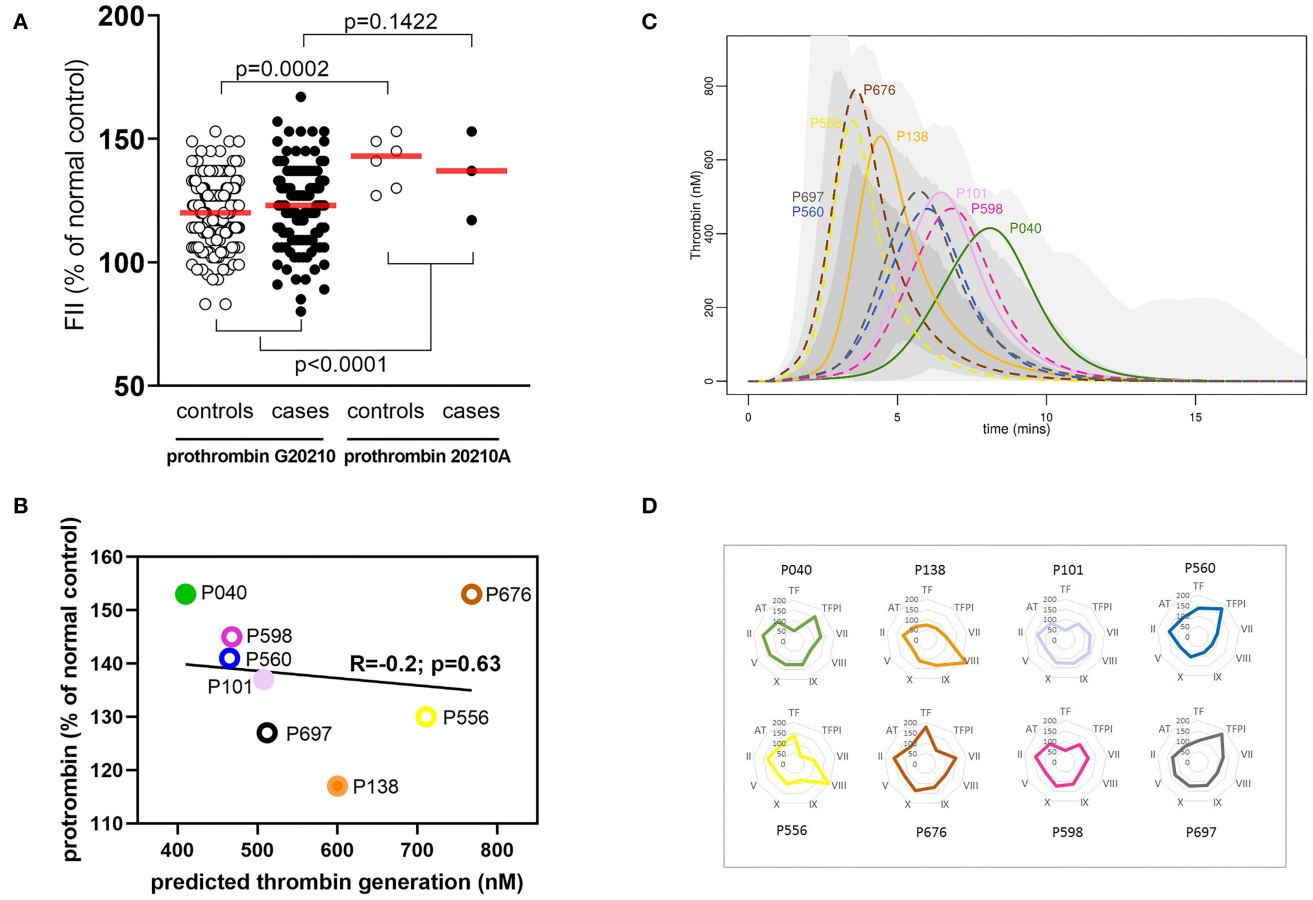
A single coagulation factor does not control the hemostatic profile. **(A)** Effect of Prothrombin variant G20210GA on plasma FII levels in pMI cases and healthy donors. Data are expressed as a percent of the reference standard, and the red bar indicates the mean for each group. **(B)** Correlation between plasma FII levels (x-axis) and predicted thrombin generation (y-axis) in plasma from individuals with the prothrombin G20210GA variant. FII levels are expressed as the percent of the reference standard. Filled symbols represent pMI cases; open symbols represent controls. **(C)** Predicted thrombin generation for the sub-group of donors with the prothrombin variant G20210A. Solid lines (case) and broken lines (control) outline the predicted rate of generation, peak activity, and depletion of thrombin for each donor. These are displayed against a shaded region that demonstrates the range of thrombin generated by the whole population of MI and control donors combined (pale gray = whole range; mid-gray = 95% quantile; and dark gray = 75% quantile). **(D)** Relative concentrations of all coagulation proteins (as the % of normal control reference standard) for individuals with the prothrombin variant G20210A. Spider plots depict the levels of each hemostatic protein for each donor. The colors used in the spider plots correspond to those used for the same donors in figures **(B,C)**.

The plasma concentration of any individual coagulation factor did not determine the overall coagulation response. To illustrate this, experimental data and model predictions were explored in the subset of donors with the prothrombin G20210A variant; this variant is chosen because it is known to affect plasma levels of FII (7). The prothrombin 20210A variant was present in 3/162 (1.85%) cases and 6/186 (3.22%) controls, in line with the reported carriage of the minor allele by <4% of the population (36). As expected, mean plasma levels of FII were elevated in individuals carrying the minor FII 20210A variant (*P* ≤ 0.0001) with levels for both cases and controls in the upper half of the normal distribution (Figure 4A; Supplementary Table SVII). However, mathematical predictions of thrombin generation varied greatly within this subset of donors and did not correlate (R = −0.2, *P* = 0.63) with plasma levels of FII (Figure 4B), implying that the level of prothrombin alone is not predictive of the overall coagulation response. As an example, two donors with similarly high plasma prothrombin levels (P040; case, and P676; control) (Figure 4B) were predicted to generate thrombin very differently (Figure 4C); rapid thrombin generation for donor P676 reached its peak at <5 min, compared to donor P040 predicted to generate less thrombin more slowly (peak thrombin >7.5 min). These differences are attributed to variation in the levels of the other coagulation proteins, resulting in different hemostatic profiles that therefore affect the overall hemostatic balance. This is illustrated in Figure 4D which shows the levels of each coagulation factor for each of these donors. So, for example, while the donor with the fastest and highest levels of thrombin generation (donor P040; pMI case) had relatively high levels of FII, this was in combination with low levels of TF and high levels of its inhibitor TFPI. Conversely, the donor with the slowest and lowest predicted thrombin generation (not donor P676; control) also had high levels of FII, but this was combined with higher levels of TF and low levels of TFPI. This analysis therefore supports the predictions of the model, suggesting that the complex interactions within the cascade mean that no single procoagulant is predictive of the overall prothrombotic potential, but is determined by the balance of the concentrations of individual procoagulant factors and inhibitors.

### Plasma Coagulation Factors in Male and Female Controls and Premature MI Patients

**Figure 5:**
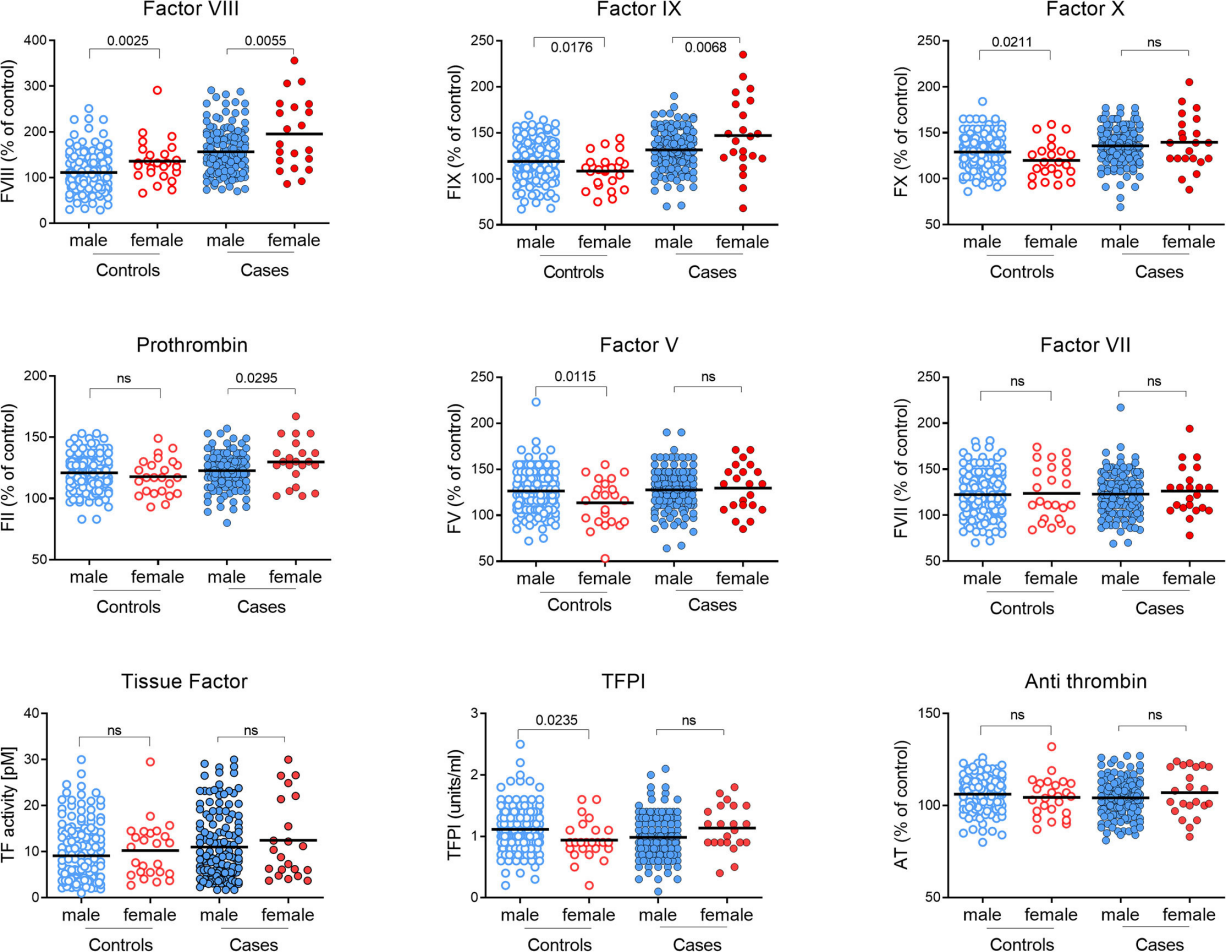
Comparison of coagulation proteins in plasma from male and female MI subjects and controls. Levels of each coagulation factor were measured in plasma from pMI cases and controls analyzed according to gender. Factors II, V, VII, VIII, IX, X, and antithrombin (AT) are reported as the percentage of reference control plasma included in each analysis, and Tissue Factor (TF) and Tissue Factor Pathway Inhibitor (TFPI) are reported as activity (pM) and (U/mL), respectively. (*n* = 139 and 23, for male and female cases; and *n* = 161 and 25 for male and female controls). (*P*-values derived by unpaired t-test after confirming data were normally distributed.

The incidence of early MI is much lower in women than in men (37, 38), and this was reflected in this dataset with only 23 of the 162 cases (14.2%) being female. Although numbers were small, female cases had significantly higher levels of prothrombin (*P* = 0.03) and FIX (*P* = 0.007) than male cases (Figure 5; Supplementary Table SVIII). In line with the existing literature (21), plasma levels of FVIII were also higher in females, in both cases and controls compared to their male counterparts. However, FVIII levels were significantly higher in female cases compared to female controls (*P* = 0.003). Although not quite reaching statistical significance, it is of note that while TFPI was lower in the cases overall, female cases had elevated levels of TFPI compared to female controls (*P* = 0.056).

### Computer Simulations of Male and Female Hemostatic Response

**Figure 6:**
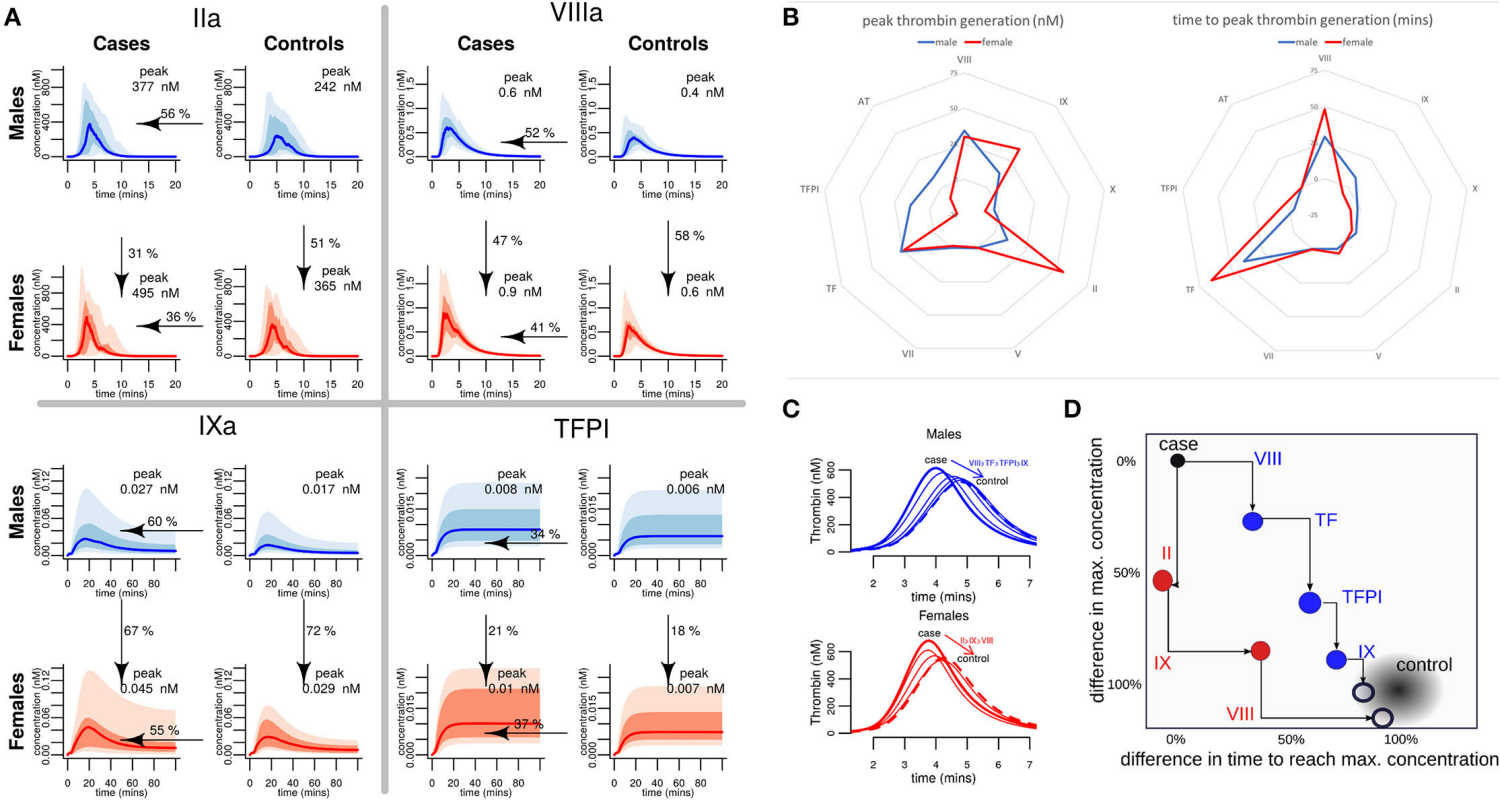
A comparison of predicted hemostatic response in male and female MI cases and healthy controls, and identification of key drivers of the differential response. **(A)** Computer predicted coagulation profiles for the proteins where the divergence between the male and the female thrombotic response was greatest. Each graph shows the median values as the bold line; shaded regions depict 95% and 75% quartiles. The median peak concentration (nM) for each factor is stated in each graph. Arrows indicate the adjustment of the median value of the data from the pMI cases (as a percentage) required to match the average (mean) value in the controls. **(B)** Assessment of the contribution of each procoagulant protein and inhibitor to the differences in the predicted peak thrombin concentration and the time to reach this peak, for males and females. **(C,D)** Demonstrate the effect on the average MI donor response of sequentially adjusting the factors with the largest difference (using the values shown arrowed in panel A) to those of controls. These factors are different for males and females. In a typical male, MI donor sequentially adjusting Factors VIII, TF, TFPI, and Factor IX brings thrombin generation close to a typical control donor. In a typical female, pMI case Factors II, IX, and VIII are sequentially adjusted to bring thrombin generation close to a typical control.

Our model simulations, based on the measured plasma coagulation factor levels, predicted that both case and control females have a larger procoagulant response than males. In healthy females, the predicted generation of thrombin was 51% greater than in healthy males (Figure 6A). Female controls were also predicted to generate more FVIIIa (47%) and FIXa (67%) and use more TFPI (21%), resulting in a profile that is similar to the predicted profiles for males who have suffered an MI at an early age. The female cases were predicted to generate 31% more thrombin than male cases, more FVIIIa (58%) and FIXa (72%), and use up more TFPI (18%), predicting a heightened procoagulant response compared to male cases (Supplementary Table SVIII and Supplementary Figures SIV–SVII).

### Key Drivers of Male and Female Coagulation Profiles

To further understand which procoagulant proteins and inhibitors drive the elevated hemostatic response predicted in male and female pMI cases, computer simulations were generated to investigate how different levels of one factor might impact the rest of the cascade. These simulations were generated using the mean plasma levels of coagulation proteins from the case cohort (assigned as a “typical case donor” profile) and compared to simulations where each coagulation protein is adjusted to its mean level in the healthy control cohort. The contribution of the plasma levels of each protein to the peak and time of thrombin generation for the “typical case donor” is illustrated in Figure 6B. This analysis shows that while the factors controlling the time required to reach peak thrombin were similar between males and females (being controlled predominantly by levels of TF and FVIII), there was a greater variation between male and female cases in the factors contributing to the maximum amount of thrombin generated (peak thrombin), suggesting that FIX and FII play a greater role in the female hemostatic response, whereas TFPI seems to play a greater role in regulating the magnitude of the response in males (Figure 6B).

To further explore the dynamics of the hemostatic profile of male and female cases compared to control, a series of simulations were produced in which the levels of coagulation factors for a “typical case donor” were sequentially adjusted to the values of a “typical control donor” (Figure 6C). We found that for a typical female case, sequentially adjusting the levels of FII, FIX, and FVIII resulted in a reduction of the predicted generation of thrombin similar to that of healthy female controls. In contrast, to return levels of thrombin generation in male premature MI cases to those of healthy male controls, adjustment of FVIII, TF, TFPI, and FIX was needed. Figure 6D illustrates the percentages and directions of these effects.

### The Cascade Is Complex and Individual Donors Show a Wide Range of Responses

**Figure 7:**
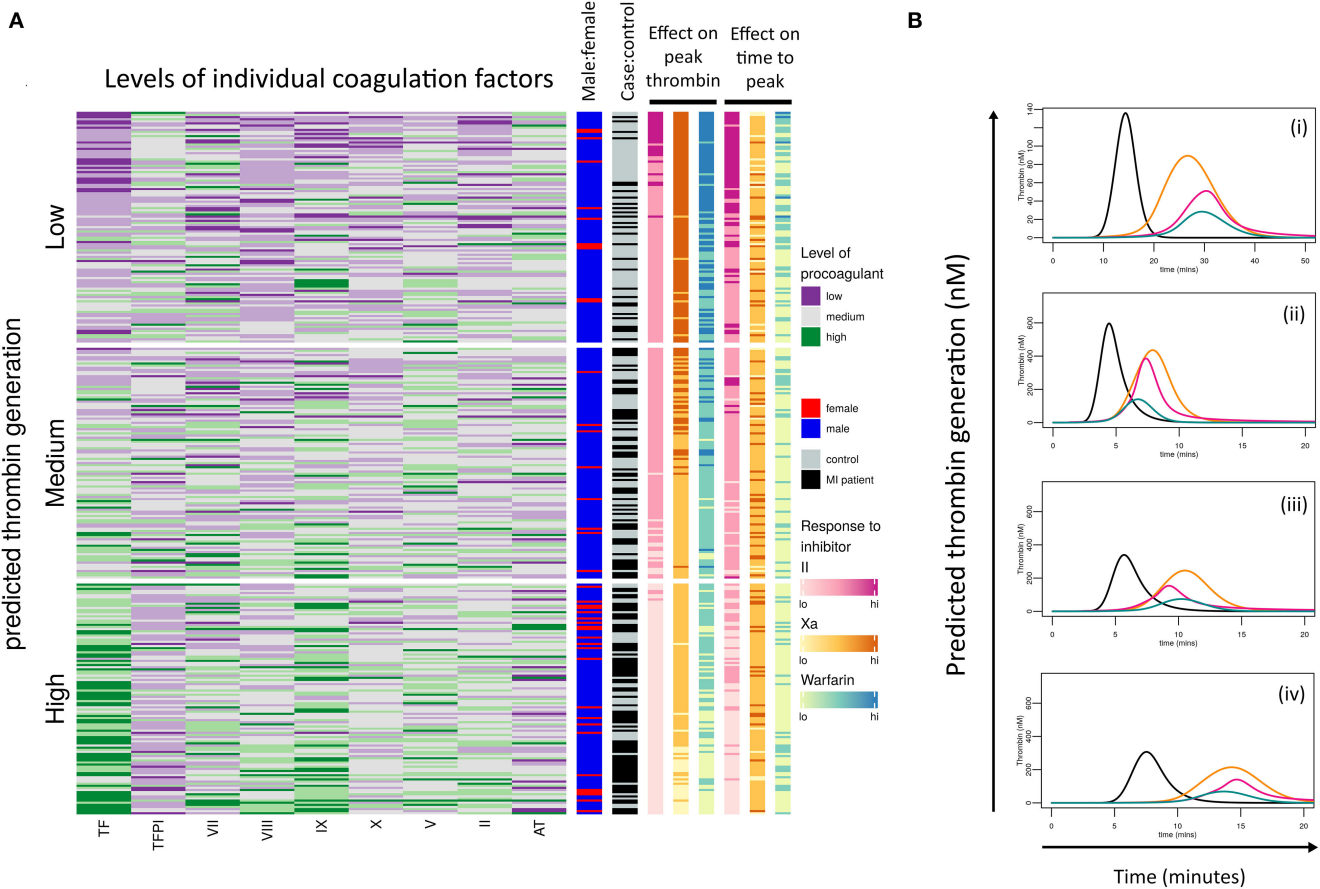
Donor response to procoagulant proteins and coagulation inhibitors is complex. **(A)** Heatmap of the calculated ability of each donor (cases and controls) to generate thrombin based on their coagulation factor profiles. Donors have been then divided into tertiles depending on their predicted ability to generate low, medium, or high concentrations of thrombin. Each row within the heatmap represents the relative levels of each procoagulant factor and inhibitor for each donor, shown as low (purple), medium (gray), or high (green). The right-hand panels (from left to right) indicate which donors are male (blue) or female (red), which are pMI cases (black) or healthy controls (gray), and the predicted effects of the anticoagulant drugs dabigatran (pink), FXa inhibitor (yellow), and warfarin (blue) on peak thrombin, and time to reach peak thrombin, respectively. **(B)** Examples of simulation plots from individual donors depicting the predicted generation of thrombin (nM) by their plasma (black line) and the predicted effect of each anticoagulant; dabigatran (pink), FXa inhibitor (orange), and warfarin (green).

Analysis was carried out to see if the model could be used for further donor stratification. The resultant heatmap (Figure 7A) is divided into tertiles based on predictions of a donor's ability to generate thrombin and the levels of each coagulation factor are also divided into tertiles and colored as follows: low = purple, medium = gray, and high = green. This highlights that donors predicted to generate high concentrations of thrombin are predominantly those who have suffered an MI (black) and that a larger proportion of the female population appears in this group (red). Many of these donors appear to have high levels of TF, FIX, and FVIII coinciding with the factors that we identified to drive the differences in responses between male donors who had suffered an early MI.

### Stratification of Donor Response to Anticoagulant Therapy

We additionally explored whether the model could be used to stratify the potential therapeutic effect of three commonly used anticoagulants that inhibit different points of the coagulation response at their standard therapeutic doses. The effects of the anticoagulants on both the simulated peak thrombin and time to peak thrombin are shown in Figure 7A in the two right-hand columns; specifically warfarin (blue; modeled by setting vitamin K dependent protein levels to 33% of their original value), the direct reversible FXa inhibitor, Rivaroxaban (orange columns; set at 6 nM), and the direct thrombin inhibitor dabigatran (pink columns; set at 0.3 μM). To provide these predictions, the mathematical model was extended and modified based on reaction schemes and values provided in Supplementary Figure SI (32, 39).

Unsurprisingly, simulations showed that in donors with low predicted levels of thrombin generation (upper tertile of heatmap), all three drugs would have a high level of efficacy in the majority of donors, whereas there was more heterogeneity in the predicted anticoagulant response as the simulated level of thrombin increased (middle and lower tertiles), with the lowest responses seen in the subjects with the highest predicted thrombin generation.

The overall inhibitory effect of each anticoagulant on both peak thrombin and time to peak was in the order: warfarin>IIa inhibitor>FXa inhibitor for all samples analyzed, reflecting the known potency of each drug at the standard therapeutic dose. However, the inhibitory effect of each drug on the onset of thrombin generation, and the time to reach peak thrombin, was more varied between donors. The representative simulated hemostatic profiles in Figure 7B show that for donor (i), dabigatran (pink line) and warfarin (blue line) were more effective at delaying the onset of thrombin generation (Figure 7Bi), whereas for donors (ii), all three inhibitors had a similar effect (Figure 7Bii). Conversely, for donor (iii), dabigatran and FXa inhibition had a similar inhibitory effect on the initiation of thrombin generation but were less effective than warfarin (Figure 7Biii), whereas dabigatran was a more effective inhibitor for delaying the onset of thrombin generation for the donor in Figure 7Biv. This donor-specific variability in the effect of different anticoagulant drugs on the hemostatic profile may be clinically relevant since each may impact the risk of forming an occlusive thrombus.

**Figure 8:**
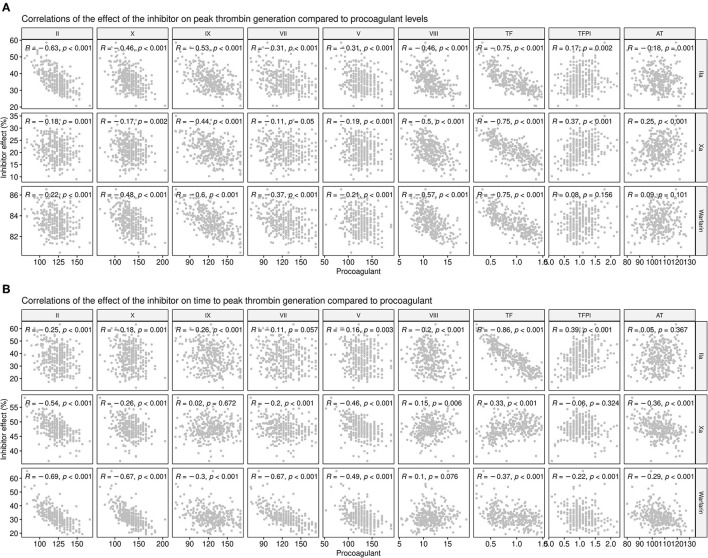
Correlations between procoagulant levels and the predicted effect of anticoagulant drugs at the standard therapeutic dose. Correlations between the measured levels of each coagulation factor (x-axes) or inhibitor with the predicted thrombin generation in the presence of anticoagulant drugs (y-axes) show the effect on **(A)** Peak thrombin and **(B)** time to reach peak thrombin. The predicted effects of the direct thrombin inhibitor, dabigatran (IIA), the FXa inhibitor, and warfarin, at standard therapeutic doses are shown as a percentage.

To explore this further, the calculated effect of the inhibitors on both the time to peak (i.e., the rate of thrombin generation) and the peak level of thrombin generated were compared to the levels of individual coagulation factors for each donor (Figure 8). While there was a reasonable degree of correlation between the predicted level of peak thrombin that would be generated in the presence of the FIIa inhibitor, FXa inhibitor, or warfarin and the measured level of TF (r = −0.75 and *P* < 0.001 for all), only the FIIa inhibitor showed a correlation with measured levels of TF on the rate of thrombin generation (time to peak; r = −0.86; *P* < 0.001). As expected, due to its broader effect on all the serine proteases, warfarin showed some degree of correlation with the measured levels of several coagulation factors, reflecting its higher potency at the standard therapeutic dose. However, while there was some correlation between the effect of the direct thrombin inhibitor on peak thrombin and the measured level of thrombin (r= −0.63; *P* < 0.001), the predicted time to reach peak thrombin levels was more closely correlated with the effect of the FXa inhibitor (r = −0.54; *P* < 0.001) and warfarin (r = −0.69; *P* < 0.001), than with FIIa inhibition (r = −0.25; *P* < 0.001). Taken together, the correlation data suggest that, while measurement of thrombin and FX levels could guide the selection of the type of direct oral anticoagulants (DOAC), patients with higher levels of circulating TF might benefit from higher doses of DOACs, therefore limiting the amplitude of the hemostatic response and potentially reducing both the risk of forming an occlusive thrombus and risk of recurrent MI.

## Discussion

While the contents of a ruptured or eroded plaque are the key initiating factors of thrombus formation during an atherothrombotic event (40), the overall hemostatic response is also important in determining the risk of ST-segment elevation MI (STEMI) from an occlusive thrombus, and thereby the risk of myocardial ischemia. Much attention has focused on platelet reactivity and the effective use of antiplatelet agents in secondary prevention, but the evidence for a causative role of platelets in MI is still relatively modest. Evidence of an association between MI and coagulation includes elevated levels of markers of thrombin generation (TAT and F1.2) in younger MI patients under clinically stable conditions (41–43), which may signal increased thrombotic events or a heightened hemostatic capacity, suggesting that targeting of specific coagulation factors may be beneficial in certain CAD patient populations. This is confirmed in recent trials investigating the combined effects of anticoagulants and antiplatelet therapy in patients with CAD (44–46), with anticoagulants now recommended for patients at heightened risk of thrombosis (47).

Our study had the dual aim of determining if patients who had suffered an MI at an early age (under 50 years) had elevated plasma levels of coagulation factors compared to well-matched healthy controls, and second, to use computer simulations to predict how these changes contribute to an enhanced prothrombotic response, and how this might be modulated by different anticoagulant drugs. The network complexity of the coagulation system renders the overall hemostatic response difficult to predict from plasma levels of individual coagulation protein levels or activity. This is illustrated by the lack of association in patients with the prothrombin 20210A variant that leads to elevated plasma levels of prothrombin, which did not predict the donor's ability to generate thrombin (Figures 4B,C). Similarly, although the patients predicted to generate high levels of thrombin tended to have higher levels of many individual factors, this was not uniformly the case, as illustrated in Figures 4D, 7.

An earlier study, modeling thrombin generation in a small cohort of donors (*n* = 28) who had acute coronary syndrome vs. stable coronary artery disease, predicted procoagulant factors II, VIII, and the inhibitor AT to be the main proteins controlling differences in thrombin generation between the two groups (48). In our larger study that compares differences between individuals who have suffered an MI at an early age, when the hemostatic risk may outweigh the atherosclerotic burden, and matched healthy donors, the modeling highlighted that FV, FVII, FVIII, and TF are rapidly depleted, and therefore limit the generation of thrombin, and FIX and FX are in excess.

Our model predicts that while the consumption of procoagulant factors and inhibitors are similar in cases and controls, propagation of the key coagulation factors that comprise the tenase and prothrombinase complexes (FXa, FIXa, and FVIIIa) are significantly different. Previously, it has been suggested that variation in levels of FXa acts as a discriminator between donors with similar profiles of thrombin generation (49). We found that, while levels of FXa varied greatly, its downstream effects were mitigated by FVa, which acts as a brake on the system.

The model allowed further stratification of the cohort by gender. The incidence of early MI is much lower in women than in men, and therefore female pMI subjects are often not studied as a separate population due to a lack of statistical power. Women are known to have elevated plasma levels of FVIII (21), which may mean that the dynamics of their hemostatic response are different from men; however, this is the first time that simulated coagulation profiles have been compared for males and females, or to compare healthy females with female pMI cases. Simulations generated using the plasma data demonstrated that in males, FVIII, FIX, TF, and TFPI (all of which regulate the earliest stages in the hemostatic cascade) are predicted to control the difference in hemostatic response between cases and controls, while in females, it is the levels of FII, FIX, and FVIII, suggesting that the key proteins that define the hemostatic profile open up the possibility that different therapeutic targets in males and females may be beneficial.

The mathematical model employed here has been used previously to provide simulations predicting the effect of the direct FXa inhibitor, Rivaroxaban, and the low molecular weight heparin, Fondaparinux (32); however, that study used a fixed concentration of TF and was based on coagulation proteins from one healthy donor. By exploring the effect of inhibitors on a large number of individuals with different hemostatic profiles we found that, while the overall inhibitory effect of each anticoagulant on peak thrombin was in the order: warfarin>IIa inhibitor>FXa inhibitor, reflecting the known potency of each drug at the standard therapeutic dose and supporting the findings of Orfeo *et al*. (32), further analysis (heatmap; Figure 7A), and correlations (Supplementary Figure SVIII) suggest that the amount of thrombin generated may be preferentially inhibited by one drug over another in individual subjects. In particular, levels of TF were strongly correlated with the predicted therapeutic potency of all three anticoagulants. The use of warfarin is gradually being superseded by DOACs which do not require regular monitoring or carry the same risks as warfarin of adverse reactions due to interactions with other drugs and clinical events. Currently, monitoring of DOAC therapy is not recommended (50), and our analysis suggests that while the levels of FII is a reasonable predictor of the magnitude of the response to a direct thrombin inhibitor, the level of FX does not predict the effect of a FXa inhibitor on either the magnitude or the rate of thrombin generation.

We recognize that this pseudo-homogeneous model has several limitations, in line with other modeling studies (24, 25, 51). First, *in vivo*, hemostasis occurs in flowing blood within a vessel wall (52, 53) and factors that are rapidly depleted in this model such as FV would be replenished from the circulation. Added to this, the model lacks platelets (52) which are a major source of FV, and other pro- and anticoagulant factors such as fibrinogen and TFPI. Platelets additionally provide the procoagulant phospholipid surface that supports coagulation protein assembly (provided by exogenous phospholipid in this study) and are also a source of polyphosphate, which provides the negative substrate that supports the activation of Factor XII *in vivo*. The model used in this study does not consider the role of FXI and FXII, key factors from the intrinsic coagulation cascade that further contribute to, and regulate, the complex dynamics of coagulation. While it could be argued that FXI activation should be incorporated, we followed the original model in excluding this interaction partly due to the high degree of variability in the rates of reactions reported in the literature, but more importantly because previous simulations showed its influence only at low concentrations of tissue factor (54). We also have not included fibrinogen in the model, for although thrombin binds to and cleaves fibrinogen, neither fibrinogen nor fibrin have been included in previous studies with this model, and attempts to include either have proved complex or are yet to be validated using real data. However, this is the largest mathematical modeling study to use measured plasma levels of coagulation factors, including endogenous TF, to generate simulations of large-scale, donor-specific, coagulation profiles for pMI and healthy cohorts, and additionally to compare the dynamics of the hemostatic response in males and females. It is increasingly recognized that MI may be under-diagnosed in women, as highlighted by major heart research organizations (e.g., American and European Heart Associations and British Heart Foundation) and other influential bodies (55–58), and therapeutic strategies developed on the basis of male-only clinical trials may be serving female MI patients poorly.

With the advent of newer direct inhibitors of coagulation factors, the possibility of stratified therapy in different patient groups becomes a possibility. Here, we illustrate that measuring thrombin, or its precursor prothrombin, or other individual coagulation factors in isolation, is not indicative of prothrombotic potential or thrombotic risk. Rather, we show that the generation of hemostatic profiles, based on a selected subset of hemostatic proteins, may be of greater value in personalized approaches to antithrombotic therapy. Further studies, on even larger cohorts are indicated, which additionally include platelets, intrinsic pathway coagulation factors, and flow, to allow further stratification of the hemostatic profile in health and disease, with the aim of rapid identification of individuals at risk of pMI and to facilitate a more personalized approach to anticoagulant therapy, which may reduce risk of secondary events.

## Data Availability Statement

The code (R and Matlab) to generate simulations from the mathematical model utilized in this paper is available via https://github.com/cardiomaths/coagSim and a version archived at the time of publication at https://doi.org/10.6084/m9.figshare.13007645.v1. An online app to run the model is available via https://cardiomaths.shinyapps.io/ThrombinGeneration/.

## Ethics Statement

The studies involving human participants were reviewed and approved by Leicestershire Health Authority Ethics Committee. The patients/participants provided their written informed consent to participate in this study.

## Author Contributions

JD, JW, and AG conceived the study and wrote the paper. JD carried out mathematical modeling, software development, and data analysis. JW carried out the laboratory analysis. NS and AG were responsible for the design and recruitment of the PRAMIS cohort and securing funding.

## Funding

JD was supported by the British Heart Foundation (RG/20/7/34866, RG/15/2/31224, PG/16/20/32074, and PG/16/36/31967) and the UK Medical Research Council (MR/W015293/1). The British Cardiac Society and British Heart Foundation (PG/01/176) supported the recruitment and laboratory analysis of the PRAMIS cohort, respectively.

## Conflict of Interest

The authors declare that the research was conducted in the absence of any commercial or financial relationships that could be construed as a potential conflict of interest.

## Publisher's Note

All claims expressed in this article are solely those of the authors and do not necessarily represent those of their affiliated organizations, or those of the publisher, the editors and the reviewers. Any product that may be evaluated in this article, or claim that may be made by its manufacturer, is not guaranteed or endorsed by the publisher.

## Abbreviations

AT: Antithrombin
DOAC: Direct oral anticoagulant
F1.2: Prothrombin fragment 1 + 2
FII/FIIa: Factor II (prothrombin)/activated Factor II (thrombin)
FV/FVa: Factor V/activated Factor V
FVII/FVIIa: Factor VII/activated Factor VII
FVIII/FVIIIa: Factor FVIII/activated Factor VIII
FIX/FIXa: Factor IX/activated Factor IX
FX/FXa: Factor X/activated Factor X
FXI: Factor XI
FXII: Factor FXII
pMI: premature MI
TAT: Thrombin antithrombin complex
TF: Tissue Factor
TFPI: Tissue Factor Pathway Inhibitor.
