# Supplementary material for: A System-Wide Investigation and Stratification of the Hemostatic Proteome in Premature Myocardial Infarction
Source: Front Cardiovasc Med. 2022 Jun 30;9:919394. doi: 10.3389/fcvm.2022.919394 (PMC9281867; doi:10.3389/fcvm.2022.919394)
Supplement: Supplementary file 1 [file Data_Sheet_1.PDF]

A system-wide investigation into stratification of the hemostatic proteome in premature Myocardial Infarction.

Joanne L Dunster, Joy R Wright, Nilesh Samani, Alison H Goodall

Contents

SI Mathematical model **S3**

SII Numerical simulations **S13**

List of Figures

SI **A schematic representing the reactions captured in the mathematical model.** The names correspond to model variables (summarised in Table SIII), with red names in boxes denoting variables with an initial condition set to a value other than zero. The arrows pointing at variables represent conversion from one species to another, broken lines denote reactions that are either introduced or had their parameter values modified in [2, 3]. Parameter names are placed next to arrows, their values are given in Table SIV. To avoid complicating the diagram the lines against the rate that FXa and IIa feedback and activate FVII (k_6 and k_7 respectively) and the reactions of AT with factors IXa, Xa, mIIa and IIa (k_{40}, k_{38}, k_{39}, k_{41} respectively) are not shown in their complete form. Underlined variables denote those adjusted to simulate the effects of Warfarin while the inhibitors Rivaroxaban and DAPA are depicted in green. S4

SII **Predictions for the depletion of procoagulants for case (solid line) and controls (broken line).** The median of numerical simulations of equations (SI.1)-(SI.4), with 5% and 95% depicted. S14

SIII **Predictions for the formation of active factors and complexes for case (solid line) and control (broken line) populations of donors.** Numerical simulations of equations (SI.1)-(SI.4). S15

SIV **Predictions for the depletion of procoagulants for males (blue) and females (red), both case (solid line) and control (broken line) populations (1 of 2).** See Figure SV for further simulations. S16

SV **Predictions for the depletion of procoagulants for males (blue) and females (red), both case (solid line) and control (broken line) populations (2 of 2).** See Figure SIV for further simulations. S17

SVI **Predictions for the formation of active factors and complexes for males (blue) and females (red), both case (solid line) and control (broken line) populations of donors (1 of 2).** See Figure SVII for further simulations. S18

SVII **Predictions for the formation of active factors and complexes for males (blue) and females (red), both case (solid line) and control (broken line) populations of donors (2 of 2).** See Figure SVI for further simulations. S19

List of Tables

SI **Reference dataset.** Normal levels of coagulation factors taken from [1]. All units are M. S4

SII **The reactions of the coagulation cascade that are captured in the mathematical model.** Reactions 1-27 constitute the original Hockin-Mann model [1] while reactions 28-29 were introduced later [2,3]. Modifications are included for interations with a reversible Factor Xa inhibitor (30-31) and a direct thrombin inhibitor (32-34). S5

SIII **Model variables.** All units are M^{-1}. A $*$ denotes vitamin K dependent protein levels that, when simulating the effects of Warfarin, are adjusted to 33% of their original value. S6

SIV **Model parameters (rates of reactions), taken from [1–3].** $*$ denotes parameters from that have been modified (in [3]) from their original values in [1]. $**$ denotes parameters newly introduced in [2,3]. Parameters below the horizontal line are those introduced for simulations of the effects of inhibitors. S7
SV **Coagulation proteins in plasma of premature MI subjects and controls.** TF, Tissue Factor; TFPI, Tissue Factor Pathway Inhibitor; AT, Anti-thrombin. Values are mean SD. Figures in bold reach statistical significance after correction for multiple testing ($p < 0.05$) S11
SVI **Predicted levels of activated factors and complexes generated in plasma from male and female premature MI subjects and controls.** TF, Tissue Factor; Values are median concentration and time to peak. S11
SVII **Comparison of coagulation proteins in plasma from donors carrying the prothrombin 202110A minor variant.** TF, Tissue Factor; TFPI, Tissue Factor Pathway Inhibitor; AT, Anti-thrombin. S12
SVIII **Comparison of coagulation proteins in plasma from male and female MI subjects and controls.** TF, Tissue Factor; TFPI, Tissue Factor Pathway Inhibitor; AT, Anti-thrombin. Values are mean SD. Figures in bold reach statistical significance after correction for multiple testing ($p < 0.05$) S12

The mathematical model detailed below has been published previously [1–4]. It is the most extensively referenced mathematical description of the coagulation cascade and has previously been used to generate profiles that reflect the hemostatic status of individuals from their individual coagulation factor composition and analyze factors that can contribute to different pathologic conditions [5–9]. Here, we include additions to the mathematical model that allow the generation of predictions of the coagulation cascade under the influence of warfarin, a direct thrombin inhibitor and a reversible factor Xa inhibitor [10–13]. Section SI provides a full description of the equations, parameters and initial conditions in addition to a description of the experimentals data. Table **??** describes the characteristics of the experimental cohort with Tables SV, SVIII and SVII providing descriptions of the experimental data, supplementing Figures 2, 4 and 5 in the main text. Numerical simulations of the mathematical model and Tables summarising their results, that supplement figures provided in the main text, are shown in Section SII.

SI Mathematical model

The reactions of the coagulation cascade that are captured in the mathematical model are summarised in Table SII and illustrated in Figure SI. The model's variables are listed in Table SIII (note we follow [1] in utilising the standard notation of square brackets employed to denote concentration) and the model's parameters are given in Table SIV. Utilising mass action kinetics the reactions of the coagulation cascade translate into a system of thirty-four coupled ordinary differential equations. The equations that describe the reactions of the extrinsic pathway are given by

$$\frac{d[TF]}{dt} = -k_2[TF]\,[VII] + k_1[TF:VII] - k_4[TF]\,[VII_a] + k_3[TF:VII_a], \tag{SI.1a}$$

$$\frac{d[VII]}{dt} = -k_2[TF]\,[VII] + k_1[TF:VII] - k_5[TF]\,[VII] - k_6[X_a]\,[VII] - k_7[II_a]\,[VII], \tag{SI.1b}$$

$$\frac{d[TF:VII]}{dt} = -k_1[TF:VII] + k_2[TF]\,[VII], \tag{SI.1c}$$

$$\frac{d[VII_a]}{dt} = k_4[TF]\,[VII_a] + k_3[TF] + k_5[TF]\,[VII] + k_6[X_a]\,[VII] + k_7[II_a]\,[VII], \tag{SI.1d}$$

$$\begin{aligned}\frac{d[TF:VII_a]}{dt} = {}& -k_3[TF:VII_a] + k_4[TF:VII_a]\,[VII_a] - k_9[TF:VII_a]\,[X] \\ & + k_8[TF:VII_a:X] - k_{12}[VII_a:TF]\,[X_a] + k_{11}[TF:VII_a:X_a] \\ & - k_{14}TF:VII_a:TF\,[IX] + k_{13}[TF:VII_a:IX] + k_{15}[TF:VII_a:IX] \\ & - k_{37}[TF:VII_a][X_a:TFPI] - k_{42}TF:VII_a\,[AT],\end{aligned} \tag{SI.1e}$$

$$\frac{d[TF:VII_a:X]}{dt} = k_9[TF]\,[X] - k_{10}[TF:VII_a:X] - k_8[TF:VII_a:X], \tag{SI.1f}$$

$$\begin{aligned}\frac{d[TF:VII_a:X_a]}{dt} = {}& k_{10}[TF:VIIa:X] + k_{12}[TF]\,[X_a] - k_{11}[TF:VIIa:Xa] \\ & - k_{36}[TF:VII_a:Xa]\,[TFPI] + k_{35}[TF:VII_a:X_a:TFPI],\end{aligned} \tag{SI.1g}$$

$$\frac{d[TF:VII_a:IX]}{dt} = k_{14}[TF]\,[IX] - k_{13}[TF:VII_a:IX] - k_{15}[TF:VII_a:IX]. \tag{SI.1h}$$

The following equations describe the reactions of the common pathway

$$\begin{aligned}\frac{d[X]}{dt} = {}& -k_9[TF:VII_a]\,[X] + k_8[TF:VIIa:X] - k_{21}[VIII_a:IX_a]\,[X] \\ & + k_{20}[VIII_a:IX_a:X] + k_{25}[VIIIa:IXa:X] - k_{43}[IX_a]\,[X],\end{aligned} \tag{SI.2a}$$

$$\frac{d[V]}{dt} = -k_{26}[II_a]\,[V] - k_{44}[mII_a]\,[V], \tag{SI.2b}$$

$$\frac{d[II]}{dt} = -k_{16}[X_a]\,[II] - k_{30}[V_a:X_a]\,[II] + k_{29}[V_a:X_a:II], \tag{SI.2c}$$

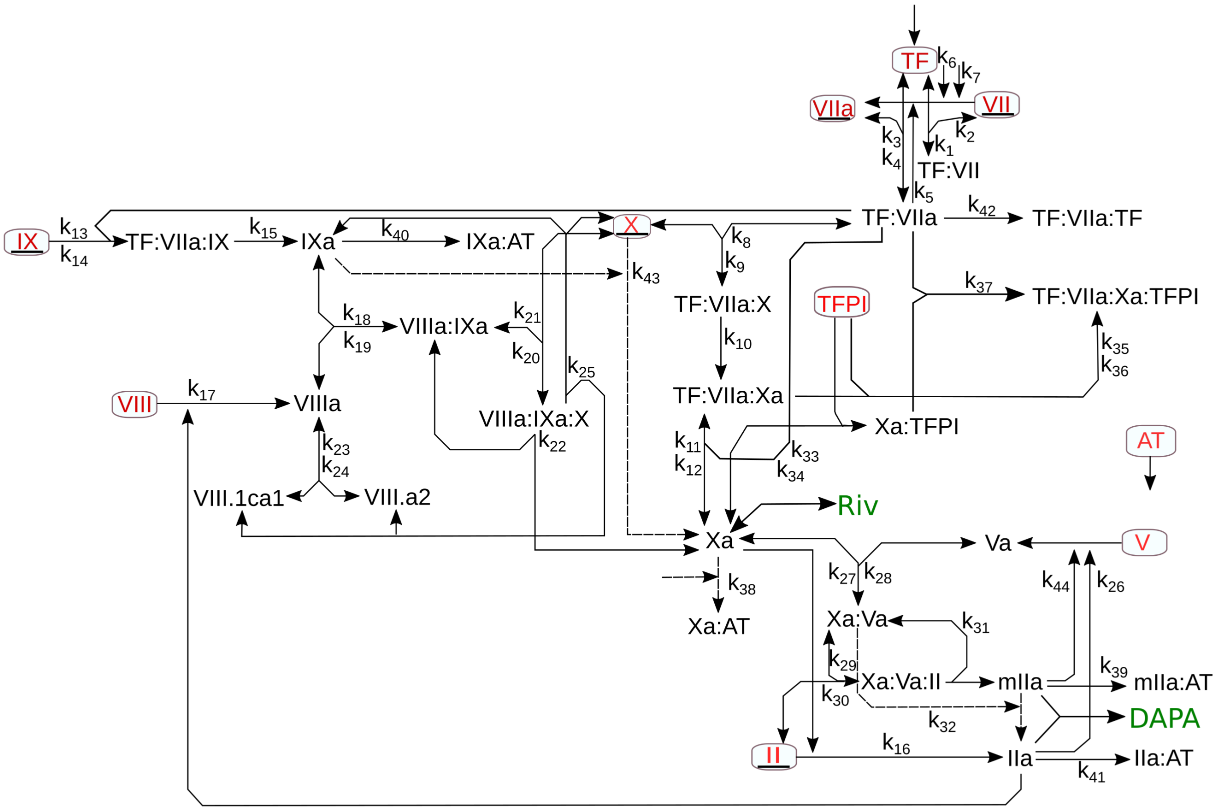

Figure SI: **A schematic representing the reactions captured in the mathematical model.** The names correspond to model variables (summarised in Table SIII), with red names in boxes denoting variables with an initial condition set to a value other than zero. The arrows pointing at variables represent conversion from one species to another, broken lines denote reactions that are either introduced or had their parameter values modified in [2, 3]. Parameter names are placed next to arrows, their values are given in Table SIV. To avoid complicating the diagram the lines against the rate that FXa and IIa feedback and activate FVII (k_6 and k_7 respectively) and the reactions of AT with factors IXa, Xa, mIIa and IIa (k_{40}, k_{38}, k_{39}, k_{41} respectively) are not shown in their complete form. Underlined variables denote those adjusted to simulate the effects of Warfarin while the inhibitors Rivaroxaban and DAPA are depicted in green.

Variable	Description	value
VII_0	Factor VII (FVII)	1.0×10^{-8}
$VIIa_0$	Activated FVII (FVIIa)	$VII_0 \times 10^{-1}$
X_0	Factor X (FX)	1.6×10^{-7}
IX_0	Factor IX (FIX)	9.0×10^{-8}
II_0	Prothrombin, factor II (FII)	1.4×10^{-6}
VIII_0	Factor VIII (FVIII)	7.0×10^{-10}
V_0	Factor V (FV)	2.5×10^{-8}
$TFPI_0$	Tissue factor pathway inhibitor (TFPI)	2.5×10^{-9}
AT_0	Antithrombin (AT)	3.4×10^{-6}

Table SI: **Reference dataset.** Normal levels of coagulation factors taken from [1]. All units are M.

Reaction	Chemical expressions
1	$\mathrm{TF{+}VII} \underset{k_1}{\overset{k_2}{\rightleftharpoons}} \mathrm{TF{:}VII}$
2	$\mathrm{TF{+}VIIa} \underset{k_3}{\overset{k_4}{\rightleftharpoons}} \mathrm{TF{:}VIIa}$
3	$\mathrm{TF{:}VIIa{+}VII} \xrightarrow{k_5} \mathrm{TF{:}VIIa{+}VIIa}$
4	$\mathrm{Xa{+}VII} \xrightarrow{k_6} \mathrm{Xa{+}VIIa}$
5	$\mathrm{IIa{+}VII} \xrightarrow{k_7} \mathrm{IIa{+}VIIa}$
6	$\mathrm{TF{:}VIIa{+}X} \underset{k_8}{\overset{k_9}{\rightleftharpoons}} \mathrm{TF{:}VIIa{:}X} \xrightarrow{k_{10}} \mathrm{TF{:}VIIa{:}Xa}$
7	$\mathrm{TF{:}VIIa{+}Xa} \underset{k_{11}}{\overset{k_{12}}{\rightleftharpoons}} \mathrm{TF{:}VIIa{:}Xa}$
8	$\mathrm{TF{:}VIIa{+}IX} \underset{k_{13}}{\overset{k_{14}}{\rightleftharpoons}} \mathrm{TF{:}VIIa{:}IX} \xrightarrow{k_{15}} \mathrm{TF{:}VIIa{+}IXa}$
9	$\mathrm{Xa{+}II} \xrightarrow{k_{16}} \mathrm{Xa{+}IIa}$
10	$\mathrm{IIa{+}VIII} \xrightarrow{k_{17}} \mathrm{IIa{+}VIIIa}$
11	$\mathrm{VIIIa{+}IXa} \underset{k_{18}}{\overset{k_{19}}{\rightleftharpoons}} \mathrm{VIIIa{:}IXa}$
12	$\mathrm{VIIIa{:}IXa{+}X} \underset{k_{20}}{\overset{k_{21}}{\rightleftharpoons}} \mathrm{VIIIa{:}IXa{:}X} \xrightarrow{k_{22}} \mathrm{VIIIa{:}IXa{+}Xa}$
13	$\mathrm{VIIIa} \underset{k_{23}}{\overset{k_{24}}{\rightleftharpoons}} \mathrm{VIIIa1{+}VIIIa2}$
14	$\mathrm{VIIIa{:}IXa{:}X} \xrightarrow{k_{25}} \mathrm{IIa{+}VIIa}$
15	$\mathrm{VIIIa{:}IXa} \xrightarrow{k_{25}} \mathrm{VIIIa1{+}VIIIa2{+}IXa}$
16	$\mathrm{IIa{+}V} \xrightarrow{k_{26}} \mathrm{IIa{+}Va}$
17	$\mathrm{Xa{+}Va} \underset{k_{27}}{\overset{k_{28}}{\rightleftharpoons}} \mathrm{Va{:}Xa}$
18	$\mathrm{Va{:}Xa{+}II} \underset{k_{29}}{\overset{k_{30}}{\rightleftharpoons}} \mathrm{Va{:}Xa{:}II} \xrightarrow{k_{31}} \mathrm{Va{:}Xa{+}mIIa}$
19	$\mathrm{mIIa{+}Va{:}Xa} \xrightarrow{k_{32}} \mathrm{IIa{+}Va{:}Xa}$
20	$\mathrm{Xa{+}TFPI} \underset{k_{33}}{\overset{k_{34}}{\rightleftharpoons}} \mathrm{Xa{:}TFPI}$
21	$\mathrm{TF{:}VIIa{+}Xa} \underset{k_{35}}{\overset{k_{36}}{\rightleftharpoons}} \mathrm{TF{:}VIIa{:}Xa{:}TFPI}$
22	$\mathrm{TF{:}VIIa{+}Xa{:}TFPI} \xrightarrow{k_{37}} \mathrm{TF{:}VIIa{:}Xa{:}TFPI}$
23	$\mathrm{Xa{+}AT} \xrightarrow{k_{38}} \mathrm{Xa{:}AT}$
24	$\mathrm{mIIa{+}AT} \xrightarrow{k_{39}} \mathrm{mIIa{:}AT}$
25	$\mathrm{IXa{+}AT} \xrightarrow{k_{40}} \mathrm{IXa{:}AT}$
26	$\mathrm{IIa{+}AT} \xrightarrow{k_{41}} \mathrm{IIa{:}AT}$
27	$\mathrm{TF{:}VIIa{+}AT} \xrightarrow{k_{42}} \mathrm{TF{:}VIIa{:}AT}$
28	$\mathrm{IXa{+}X} \xrightarrow{k_{43}} \mathrm{IXa{+}Xa}$
29	$\mathrm{mIIa{+}V} \xrightarrow{k_{44}} \mathrm{mIIa{+}Va}$
30	$\mathrm{Xa{+}Riv} \xrightarrow{r_1} \mathrm{Xa{:}Riv}$
31	$\mathrm{Xa{:}Va{+}Riv} \xrightarrow{r_3} \mathrm{Xa{:}Va{:}Riv}$
32	$\mathrm{IIa{+}DAPA} \xrightarrow{d_1} \mathrm{IIa{:}DAPA}$
33	$\mathrm{mIIa{+}DAPA} \xrightarrow{d_3} \mathrm{mIIa{:}DAPA}$

Table SII: **The reactions of the coagulation cascade that are captured in the mathematical model.** Reactions 1-27 constitute the original Hockin-Mann model [1] while reactions 28-29 were introduced later [2,3]. Modifications are included for interations with a reversible Factor Xa inhibitor (30-31) and a direct thrombin inhibitor (32-34).

Variable	Description	Initial condition
$[TF]$	Tissue factor	patient data
$[VII]$	Factor VII	patient data $*$
$[TF:VII]$	FVII bound to TF	0
$[VIIa]$	Activated FVII	patient data $*$
$[TF:VIIa]$	Extrinsic tenase, FVIIa bound to TF	0
$[X]$	Factor X	patient data $*$
$[Xa]$	Activated FX	0
$[TF:VIIa:X]$	TF:VIIa bound to X	0
$[TF:VIIa:Xa]$	TF:VIIa bound to Xa	0
$[IX]$	Factor IX	patient data $*$
$[IXa]$	Activated FIX	0
$[TF:VIIa:IX]$	TF:VIIa bound to IX	0
$[II]$	Prothrombin, factor II	patient data $*$
$[IIa]$	Thrombin, activated FII	0
$[VIII]$	Factor VIII	patient data
$[VIIIa]$	Activated FVIII	0
$[VIIIa:IXa]$	Intrinsic tenase, FVIIIa bound to IXa	0
$[VIIIa:IXa:X]$	Intrinsic tenase bound to FX	0
$[VIIIa1]$	FVIIIa1	0
$[VIIIa2]$	FVIIIa2	0
$[V]$	Factor V	patient data
$[Va]$	Activated FV	0
$[Va:Xa]$	Prothrombinase, FVa bound to Xa	0
$[Va:Xa:II]$	Prothrombinase bound to prothrombin	0
$[mIIa]$	Mesothrombin, activated FII	0
$[TFPI]$	Tissue factor pathway inhibitor	patient data
$[TFPI:Xa]$	TFPI bound to FXa	0
$[TF:VIIa:TFPI:Xa]$	TF:VIIa bound to TFPI:Xa	0
$[AT]$	Antithrombin	patient data
$[Xa:AT]$	AT bound to Xa	0
$[mIIa:AT]$	AT bound to mIIa	0
$[IXa:AT]$	AT bound to IXa	0
$[IIa:AT]$	AT bound to IIa	0
$[TF:VIIa:AT]$	AT bound to TF:VIIa	0
$[Riv]$	Rivaroxaban	0 or $4e-9$
$[Xa:Riv]$	FXa bound to Rivaroxaban	0
$[Xa:Va:Riv]$	Prothrombinase bound to Rivaroxaban	0
$[DAPA]$	DAPA	0 or $3e-7$
$[IIa:DAPA]$	Thrombin bound to DAPA	0
$[mIIa:DAPA]$	mIIa bound to DAPA	0

Table SIII: **Model variables.** All units are M^{-1}. A $*$ denotes vitamin K dependent protein levels that, when simulating the effects of Warfarin, are adjusted to 33% of their original value.

Parameter	Units	Value	Parameter	Units	Value
k_1	s^{-1}	3.1×10^{-3}	k_{23}	$M^{-1}s^{-1}$	2.2×10^4
k_2	$M^{-1}s^{-1}$	3.2×10^6	k_{24}	s^{-1}	6×10^{-3}
k_3	s^{-1}	3.1×10^{-3}	k_{25}	s^{-1}	1×10^{-3}
k_4	$M^{-1}s^{-1}$	2.3×10^7	k_{26}	$M^{-1}s^{-1}$	2×10^7
k_5	s^{-1}	4.4×10^5	k_{27}	s^{-1}	0.2
k_6	$M^{-1}s^{-1}$	1.3×10^7	k_{28}	$M^{-1}s^{-1}$	4×10^8
k_7	$M^{-1}s^{-1}$	2.3×10^4	k_{29}	s^{-1}	103
k_8	s^{-1}	1.05	k_{30}	$M^{-1}s^{-1}$	1×10^8
k_9	$M^{-1}s^{-1}$	2.5×10^7	k_{31}	s^{-1}	63.5
k_{10}	s^{-1}	6	$* \, k_{32}$	$M^{-1}s^{-1}$	2.3×10^8
k_{11}	s^{-1}	19	k_{33}	s^{-1}	3.6×10^{-4}
k_{12}	$M^{-1}s^{-1}$	2.2×10^7	k_{34}	$M^{-1}s^{-1}$	9×10^5
k_{13}	s^{-1}	2.4	k_{35}	s^{-1}	1.1×10^{-4}
k_{14}	$M^{-1}s^{-1}$	1×10^7	k_{36}	$M^{-1}s^{-1}$	3.2×10^8
k_{15}	s^{-1}	1.8	k_{37}	$M^{-1}s^{-1}$	5×10^7
k_{16}	$M^{-1}s^{-1}$	7.5×10^3	$* \, k_{38}$	$M^{-1}s^{-1}$	4.2×10^3
k_{17}	$M^{-1}s^{-1}$	2×10^7	k_{39}	$M^{-1}s^{-1}$	7.1×10^3
k_{18}	s^{-1}	5×10^{-3}	k_{40}	$M^{-1}s^{-1}$	4.9×10^2
k_{19}	$M^{-1}s^{-1}$	1×10^7	k_{41}	$M^{-1}s^{-1}$	7.1×10^3
k_{20}	s^{-1}	1×10^{-3}	k_{42}	$M^{-1}s^{-1}$	2.3×10^2
k_{21}	$M^{-1}s^{-1}$	1×10^8	$** \, k_{43}$	$M^{-1}s^{-1}$	5.7×10^3
k_{22}	s^{-1}	8.2	$** \, k_{44}$	$M^{-1}s^{-1}$	3×10^6
r_1	$M^{-1}s^{-1}$	1×10^8	d_1	$M^{-1}s^{-1}$	2.5×10^6
r_2	s^{-1}	4×10^{-2}	d_2	s^{-1}	5×10^{-2}
r_3	s^{-1}	2.1×10^{-1}	d_3	$M^{-1}s^{-1}$	8.5×10^4

Table SIV: **Model parameters (rates of reactions), taken from [1–3].** $*$ denotes parameters from that have been modified (in [3]) from their original values in [1]. $**$ denotes parameters newly introduced in [2, 3]. Parameters below the horizontal line are those introduced for simulations of the effects of inhibitors.

$$\frac{d[X_a]}{dt} = -k_{12}[VII_a : TF]\,[X_a] + k_{11}[VII_a : TF : X_a] - k_{28}[X_a]\,[V_a] + k_{27}[V_a : X_a] + k_{22}[VIII_a : IX_a : X] - k_{34}[X_a][TFPI] + k_{33}[X_a : TFPI] - k_{38}[X_a]\,[AT] + k_{43}[IX_a]\,[X] - r_1[X_a]\,[Riv] + r_2[X_a : Riv], \quad \text{(SI.2d)}$$

$$\frac{d[V_a]}{dt} = k_{26}[II_a]\,[V] - k_{28}[X_a]\,[V_a] + k_{27}[V_a : X_a] + k_{44}[mII_a]\,[V], \quad \text{(SI.2e)}$$

$$\frac{d[V_a : X_a]}{dt} = k_{28}[X_a]\,[V_a] - k_{27}[V_a : X_a] - k_{30}[V_a : X_a]\,[II] + k_{29}[V_a : X_a : II] + k_{31}[V_a : X_a : II] - r_1\,[V_a : X_a]\,[Riv] + r_3[V_a : X_a : Riv], \quad \text{(SI.2f)}$$

$$\frac{d[V_a : X_a : II]}{dt} = k_{30}[V_a : X_a]\,[II] - k_{29}[V_a : X_a : II] - k_{31}[V_a : X_a : II], \quad \text{(SI.2g)}$$

$$\frac{d[II_a]}{dt} = k_{16}[X_a]\,[II] + k_{32}[mII_a] : [V_a : X_a] - k_{41}[II_a]\,[AT], \quad \text{(SI.2h)}$$

$$\frac{d[mII_a]}{dt} = k_{31}[V_a : X_a : II] - k_{32}[mII_a]\,[Va : Xa] - k_{39}[mII_a]\,[AT], \quad \text{(SI.2i)}$$

and the reactions of the intrinsic pathway are given by

$$\frac{d[IX]}{dt} = -k_{14}[TF : VII_a\,[IX] + k_{13}[VII_a : TF : IX], \quad \text{(SI.3a)}$$

$$\frac{d[VIII]}{dt} = -k_{17}[II_a]\,[VIII], \quad \text{(SI.3b)}$$

$$\frac{d[IX_a]}{dt} = k_{15}[TF : VII_a : IX] - k_{19}[VIII_a]\,[IX_a] + k_{18}[VIII_a : IX_a] + k_{25}[VIII_a : IX_a : X] + k_{25}[VIII_a : IX_a] - k_{40}[IX_a]\,[AT], \quad \text{(SI.3c)}$$

$$\frac{d[VIII_a]}{dt} = k_{17}[II_a]\,[VIII] - k_{19}[VIII_a]\,[IX_a] + k_{18}[VIII_a : IX_a] - k_{24}[VIII_a] + k_{23}[VIII1_a]\,[VIII2_a], \quad \text{(SI.3d)}$$

$$\frac{d[VIII1_a]}{dt} = k_{24}[VIII_a] + k_{25}[VIII_a : IX_a : X] + k_{25}[VIII_a : IX_a] - k_{23}[VIII1_a][VIII2_a], \quad \text{(SI.3e)}$$

$$\frac{d[VIII2_a]}{dt} = k_{24}[VIII_a] + k_{25}[VIII_a : IX_a : X] + k_{25}[VIII_a : IX_a] - k_{23}[VIII1_a][VIII2_a], \quad \text{(SI.3f)}$$

$$\frac{d[VIII_a : IX_a]}{dt} = k_{19}[VIII_a]\,[IX_a] - k_{18}[VIII_a : IX_a] - k_{21}[VIII_a : IX_a]\,[X] + k_{20}[VIII_a : IX_a : X] + k_{22}[VIII_a : IX_a : X] - k_{25}[VIII_a : IX_a], \quad \text{(SI.3g)}$$

$$\frac{d[VIII_a : IX_a : X]}{dt} = k_{21}[VIII_a : IX_a]\,[X] - k_{20}[VIII_a : IX_a : X] - k_{22}[VIII_a IX_a : X] - k_{25}[VIIIa : IXa : X]. \quad \text{(SI.3h)}$$

The model incorporates two inhibitors, the equations that capture the interactions with the inhibitor TFPI are given by

$$\frac{d[TFPI]}{dt} = -k_{34}[X_a]\,[TFPI] + k_{33}[X_a : TFPI] - k_{36}[TF : VII_a : X_a]\,[TFPI] + k_{35}[TF : VII_a : X_a : TFPI], \quad \text{(SI.4a)}$$

$$\frac{d[X_a : TFPI]}{dt} = k_{34}[X_a]\,[TFPI] - k_{33}[X_a : TFPI] - k_{37}[TF]\,[X_a : TFPI], \quad \text{(SI.4b)}$$

$$\frac{d[TF : VII_a : X_a : TFPI]}{dt} = k_{36}[TF : VII_a : X_a]\,TFPI - k_{35}[TF : VII_a : X_a : TFPI] + k_{37}[TF : VII_a]\,[X_a : TFPI], \quad \text{(SI.4c)}$$

and those that describe ATIII interactions are given by

$$\frac{d[AT]}{dt} = -k_{38}[X_a]\,[AT] - k_{39}[mII_a]\,[AT] - k_{40}[IX_a]\,[AT] - k_{41}[II_a]\,[AT] - k_{42}[TF:VII_a]\,[AT], \tag{SI.5a}$$

$$\frac{[Xa:AT]}{dt} = k_{38}[X_a]\,[AT], \tag{SI.5b}$$

$$\frac{[mII_a:AT]}{dt} = k_{39}[mII_a]\,[AT], \tag{SI.5c}$$

$$\frac{[IX_a:AT]}{dt} = k_{40}[IX_a]\,[AT], \tag{SI.5d}$$

$$\frac{[II_a:AT]}{dt} = k_{41}[II_a]\,[AT], \tag{SI.5e}$$

$$\frac{[TF:VII_a:AT]}{dt} = k_{42}[TF:VII_a]\,[AT]. \tag{SI.5f}$$

The equations for the binding of Rivaroxaban with factor Xa and prothrombinase are

$$\frac{d[Riv]}{dt} = -r_1[X_a]\,[Riv] + r_2[X_a:Riv] - r_1[X_a:V_a]\,[Riv] + r_3[X_a:V_a:Riv], \tag{SI.5g}$$

$$\frac{d[X_a:Riv]}{dt} = r_1[X_a]\,[Riv] - r_2[X_a:Riv], \tag{SI.5h}$$

$$\frac{d[X_a:V_a:Riv]}{dt} = r_1[X_a:V_a]\,[Riv] - r_3[X_a:V_a:Riv], \tag{SI.5i}$$

$$\tag{SI.5j}$$

and the equations for the binding of Dapa with thrombin and mesothrombin are given by

$$\frac{d[DAPA]}{dt} = -d_1[II_a]\,[DAPA] + d_2[II_a:DAPA] - d_3[mII_a]\,[DAPA] + d_2[mII_a:DAPA], \tag{SI.5k}$$

$$\frac{d[II_a:DAPA]}{dt} = d_1[II_a]\,[DAPA] - d_2[II_a:DAPA], \tag{SI.5l}$$

$$\frac{d[mII_a:DAPA]}{dt} = d_3[mII_a]\,[DAPA] - d_2[mII_a:DAPA]. \tag{SI.5m}$$

$$\tag{SI.5n}$$

The initial conditions (described in Table SIII) of all procoagulants and inhibitors are set to values measured in plasma obtained from human donors while the initial conditions of downstream activated and inactivated factors are set to zero. i.e. at $t = 0$ we set $[TF] = TF_0$, $[II] = II_0$, $[V] = V_0$, $[VII] = VII_0$, $[VIIa] = VII_0$, $[VIII] = VIII_0$, $[IX] = IX_0$, $[X] = X_0$, $[TFPI] = TFPI_0$, $[AT] = AT_0$ and $[TF:VII] = [TF:VIIa] = [Xa] = [TF:VIIa:X] = [TF:VIIa:Xa] = [IXa] = [TF:VIIa:IX] = [IIa] = [VIIIa] = [VIIIa:IXa] = [VIIIa:IXa:X] = [VIIIa1] = [VIIIa2] = [Va] = [Va:Xa] = [Va:Xa:II] = [mIIa] = [TFPI:Xa] = [TF:VIIa:TFPI:Xa] = [Xa:AT] = [mIIa:AT] = [IXa:AT] = [IIa:AT] = [TF:VIIa:AT] = [DAPA] = [Riv] = 0$.

For simulations of the cascade under the effects of Warfarin we set $[X_0] = 0.33\,X_0$, $[II_0] = 0.33\,II_0$, $[IX_0] = 0.33\,IX_0$, $[VII_0] = 0.33\,VII_0$, $[VIIa_0] = 0.33\,VIIa_0$, for Rivaroxaban $[Riv] = [Riv_0]$ and for DAPA $[DAPA] = DAPA_0$.

We note that in this system we have conservation (in their various forms) of tissue factor, factors II, V, VII,

VIII, IX and X and the inhibitors TFPI and AT as expressed by

$$
\begin{aligned}
TF_0 &= [TF] + [TF:VII] + [TF:VIIa] + [TF:VIIa:X] + [TF:VIIa:Xa] \\
&\quad + [TF:VIIa:IX] + [TF:VIIa:Xa:TFPI] + [TF:VIIa:AT], && \text{(SI.6)} \\
II_0 &= [II] + [Va:Xa:II] + [IIa] + [mIIa] + [mIIa:AT] + [IIa:AT], && \text{(SI.7)} \\
V_0 &= [V] + [Va] + [Va:Xa] + [Va:Xa:II], && \text{(SI.8)} \\
VII_0 &= [VII] + [TF:VII] + [VIIa] + [TF:VIIa] + [TF:VIIa:X] \\
&\quad + [TF:VIIa:Xa] + [TF:VIIa:IX] + [TF:VIIa:AT], && \text{(SI.9)} \\
VIII_0 &= [VIII] + [VIIIa] + [VIII1a] + [VIII2a] + [VIIIa:IXa] \\
&\quad + [VIIIa:IXa:X], && \text{(SI.10)} \\
IX_0 &= [IX] + [TF:VIIa:IX] + [IXa] + [VIIIa:IXa] + [VIIIa:IXa:X] \\
&\quad + [IXa:AT], && \text{(SI.11)} \\
X_0 &= [X] + [TF:VIIa:X] + [TF:VIIa:Xa] + [Xa] + [Va:Xa] \\
&\quad + [Va:Xa:II] + [Xa:AT], && \text{(SI.12)} \\
TFPI_0 &= [TFPI] + [Xa:TFPI] + [TF:VIIa:Xa:TFPI], && \text{(SI.13)} \\
AT_0 &= [AT] + [Xa:AT] + [mIIa:AT] + [IXa:AT] + [IIa:AT] \\
&\quad + [TF:VIIa:AT], && \text{(SI.14)}
\end{aligned}
$$

which follow from (SI.1)-(SI.5).

Coagulation Protein	Cases	Controls	p-value
TF[pM]	11.32 ± 7.7	9.27 ± 6.4	**0.0070**
FII[%]	123 ± 14.5	121 ± 13.6	0.0532
FV[%]	127 ± 22.5	125 ± 22.8	0.2749
FVII[%]	123 ± 23.2	123 ± 23.7	0.8518
FVIII[%]	161 ± 55.6	115 ± 41.4	$<$ **0.0001**
FIX[%]	133 ± 25.6	118 ± 20.7	$<$ **0.0001**
FX[%]	136 ± 21.5	128 ± 18.0	**0.0003**
TFPI[Units/mL]	1.008 ± 0.4	1.090 ± 0.4	**0.0330**
ATIII[%]	104 ± 10.7	106 ± 8.7	0.1212

Table SV: **Coagulation proteins in plasma of premature MI subjects and controls.** TF, Tissue Factor; TFPI, Tissue Factor Pathway Inhibitor; AT, Anti-thrombin. Values are mean SD. Figures in bold reach statistical significance after correction for multiple testing ($p < 0.05$)

Coagulation Product	Cases		Controls	
	Male	Female	Male	Female
IIa	377 nM	495 nM	242 nM	365 nM
	4 min	5 min	5 min	4 min
Va	25 nM	25 nM	24 nM	24 nM
	3 min	3 min	3 min	3 min
VIIa	12 nM	13 nM	12 nM	12 nM
	75 min	92 min	67 min	21 min
VIIIa	0.6 nM	0.9 nM	0.4 nM	0.6 nM
	3 min	2 min	4 min	3 min
IXa	0.03	0.04	0.02 nM	0.03
	16 min	19 min	16 min	17 min
Xa	1.2 nM	2.6 nM	0.3 nM	1.3 nM
	23 min	19 min	27 min	21 min
TF:VIIa	0.0004 nM	0.00005 nM	0.00003 nM	0.00005 nM
	5 min	4 min	6 min	5 min
Va:Xa	18 nM	21 nM	10 nM	16 nM
	23 min	18 min	25 min	20 min
VIIa:IXa	0.006 nM	0.009 nM	0.003 nM	0.006 nM
	9 min	9 min	10 min	9 min
VIIIo	2.09 nM	2.41 nM	1.44 nM	1.84 nM
	92 min	64 min	64 min	109 min

Table SVI: **Predicted levels of activated factors and complexes generated in plasma from male and female premature MI subjects and controls.** TF, Tissue Factor; Values are median concentration and time to peak.

Donor	Gender	FII [%]	FV [%]	FVII [%]	FX [%]	FVIII [%]	FIX [%]	ATIII [%]	TF [pM]	TFPI [Units/mL]
P040	F	153	134	130	122	92	123	124	4.7	1.7
P101	M	137	106	126	122	133	123	106	4.3	1.1
P138	M	117	82	94	111	223	132	88	6.7	0.8
556	F	130	93	96	105	190	86	109	12.5	0.5
560	M	141	102	94	105	78	82	117	12.8	1.9
598	M	145	106	111	122	89	112	115	5.3	1.2
676	M	153	116	147	139	111	123	109	16.5	0.9
697	M	127	128	122	122	107	121	100	9.6	1.9

Table SVII: **Comparison of coagulation proteins in plasma from donors carrying the prothrombin 202110A minor variant.** TF, Tissue Factor; TFPI, Tissue Factor Pathway Inhibitor; AT, Anti-thrombin.

	Cases			Controls		
Coagulation Protein	Male	Female	p-value	Male	Female	p-value
TF[pM]	11.0 ± 7.5	12.5 ± 8.7	0.4010	9.12 ± 6.4	10.26 ± 6.1	0.4061
FII[%]	**123 ± 13.4**	**130 ± 17.9**	**0.0295**	121 ± 13.5	118 ± 14.7	0.2884
FV[%]	128 ± 21.5	130 ± 25.8	0.6837	126 ± 22.1	114 ± 25.2	0.0118
FVII[%]	123 ± 22.4	126 ± 26.4	0.5134	122 ± 22.8	124 ± 29.6	0.8062
FVIII[%]	**156 ± 49.8**	**195 ± 77.7**	**0.0025**	**111 ± 39.6**	**136 ± 46.7**	**0.0055**
FIX[%]	**131 ± 21.6**	**147 ± 41.0**	0.0067	119 ± 20.8	108 ± 18.0	0.0177
FX[%]	136 ± 19.7	140 ± 28.8	0.4361	129 ± 17.5	120 ± 19.8	0.0211
TFPI[Units/mL]	0.98 ± 0.4	1.14 ± 0.4	0.0623	1.11 ± 0.4	0.94 ± 0.3	0.0235
ATIII[%]	104 ± 10.1	107 ± 12.7	0.2397	106 ± 8.5	104 ± 10.4	0.3535

Table SVIII: **Comparison of coagulation proteins in plasma from male and female MI subjects and controls.** TF, Tissue Factor; TFPI, Tissue Factor Pathway Inhibitor; AT, Anti-thrombin. Values are mean SD. Figures in bold reach statistical significance after correction for multiple testing ($p < 0.05$)

SII Numerical simulations

The model is solved numerically using parameter values fixed to those in Table SIV. Procoagulant and inhibitor levels measured in plasma taken from individual donors form model inputs (initial conditions). The range of these inputs can be converted to concentrations by multiplying by the normal range of concentrations measured in a reference set of donors [1]. Model simulations yield a set of thirty-four time-dependent profiles for each donor. These are displayed as procoagulants (Factors II, V, VII, VIII, IX, X, TF, TFPI and AT), that are depleted, downstream activated factors (IIa, Va, VIIa, VIIIa, IXa, Xa) and complexes (TF:VIIa, Va:Xa, VIIIa:IXa, VIIIo). While the majority of these procoagulants, factors or complexes are the output of a single model variable (i.e. the predicted changes in the procoagulant Factor V are obtained from the numerical solutions to equation (SI.2b)) the following are composites of multiple model variables:

$$IIa = [IIa] + [mIIa], \tag{SII.1}$$

$$TF = [TF] + [TF:VII], \tag{SII.2}$$

$$\begin{aligned} TF:VIIa = [TF:VIIa] + [TF:VIIa:X] + [TF:VIIa:Xa] \\ + [TF:VIIa:IX], \end{aligned} \tag{SII.3}$$

$$VIIIa:IXa = [VIIIa:IXa] + [VIIIa:IXa:X], \tag{SII.4}$$

$$Va:Xa = [Va:Xa] + [Va:Xa:II], \tag{SII.5}$$

$$VIIIo = [VIIIa1] + [VIIIa2]. \tag{SII.6}$$

Note that due to consertvation all thirty-four model variables are accounted for in the reported simulations.

The median levels (and 5%, 25%, 75% and 95% quantiles) of the concentrations acheived across populations, such as case and control cohorts, are calculated at one second intervals. Computer predictions of the time-dependent responses in the median of case and control donors are shown in Figures SII and SIII. Simulations highlighting the differences in the predictions split between male and female case and control donors are shown in Figures SIV, SV, SVI and SVII.

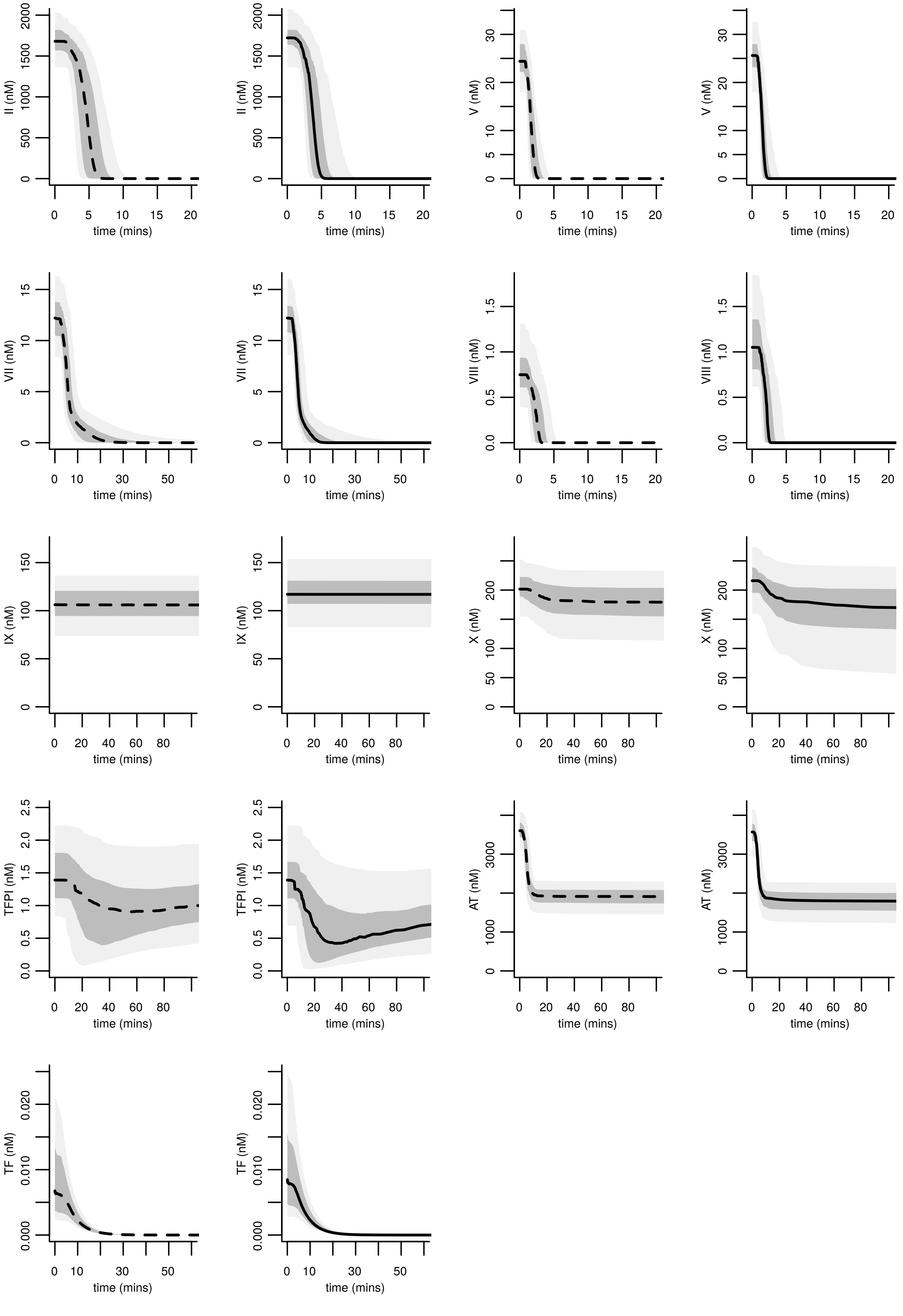

Figure SII: **Predictions for the depletion of procoagulants for case (solid line) and controls (broken line).** The median of numerical simulations of equations (SI.1)-(SI.4), with 5% and 95% depicted.

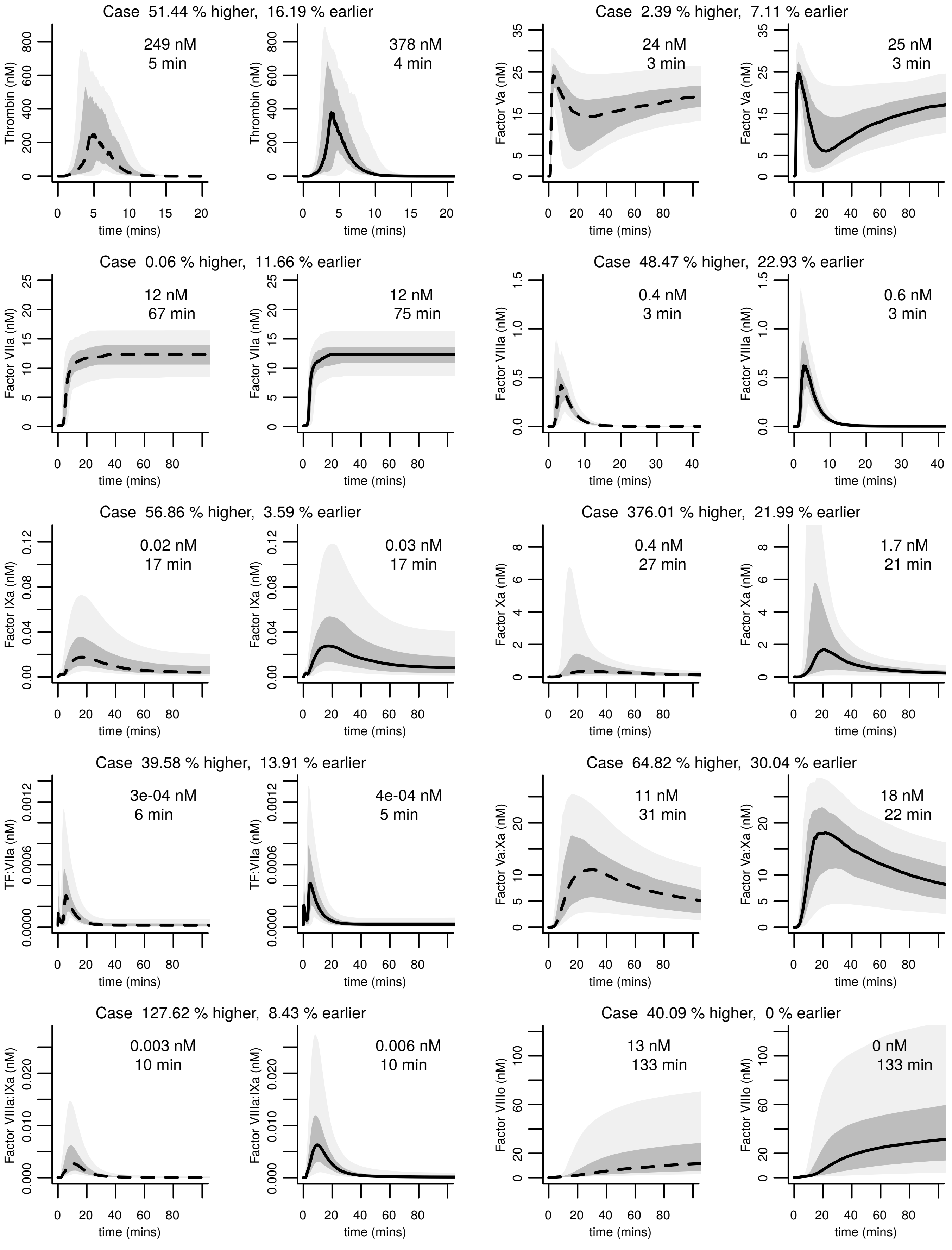

Figure SIII: **Predictions for the formation of active factors and complexes for case (solid line) and control (broken line) populations of donors.** Numerical simulations of equations (SI.1)-(SI.4).

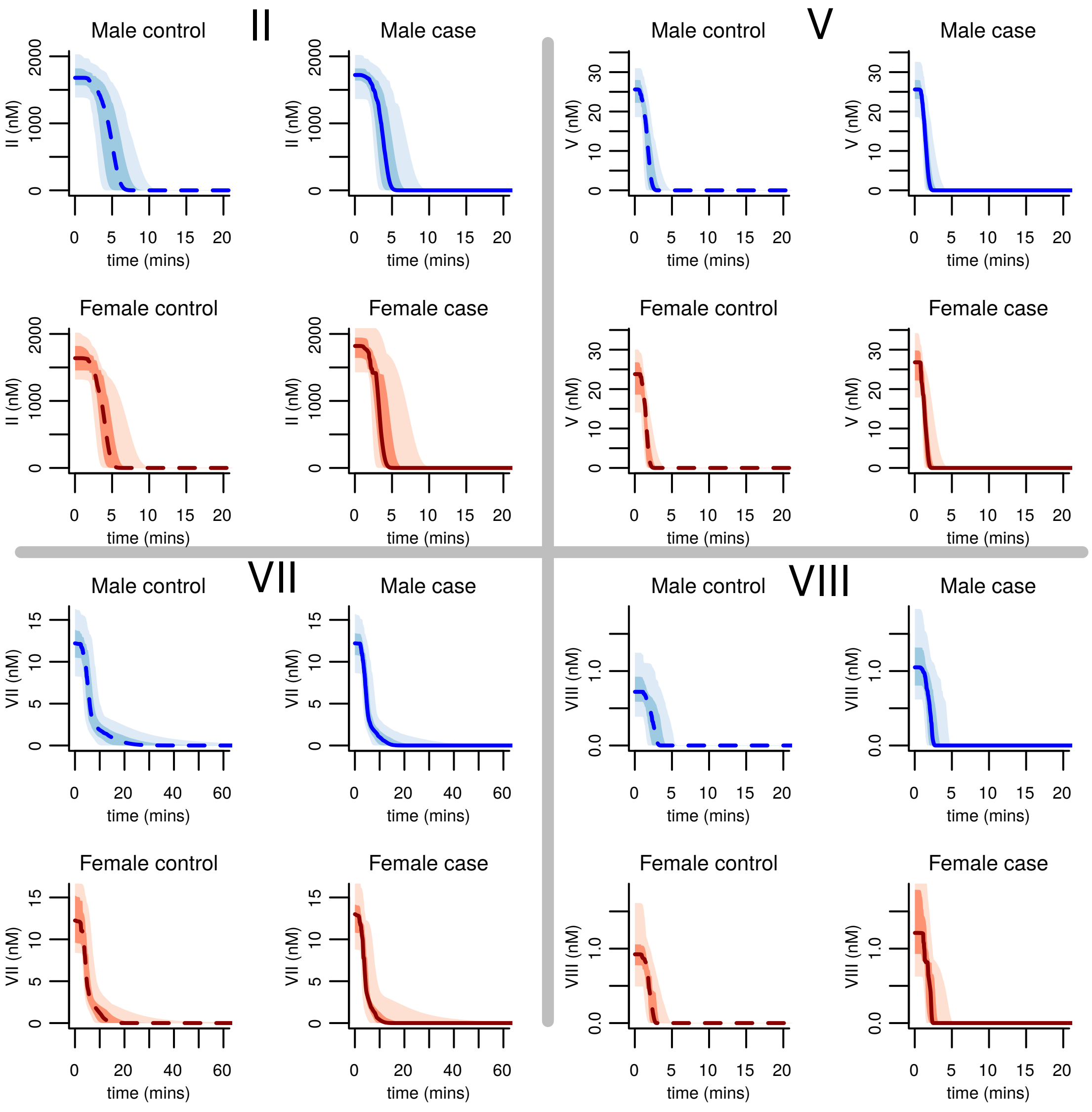

Figure SIV: **Predictions for the depletion of procoagulants for males (blue) and females (red), both case (solid line) and control (broken line) populations (1 of 2).** See Figure SV for further simulations.

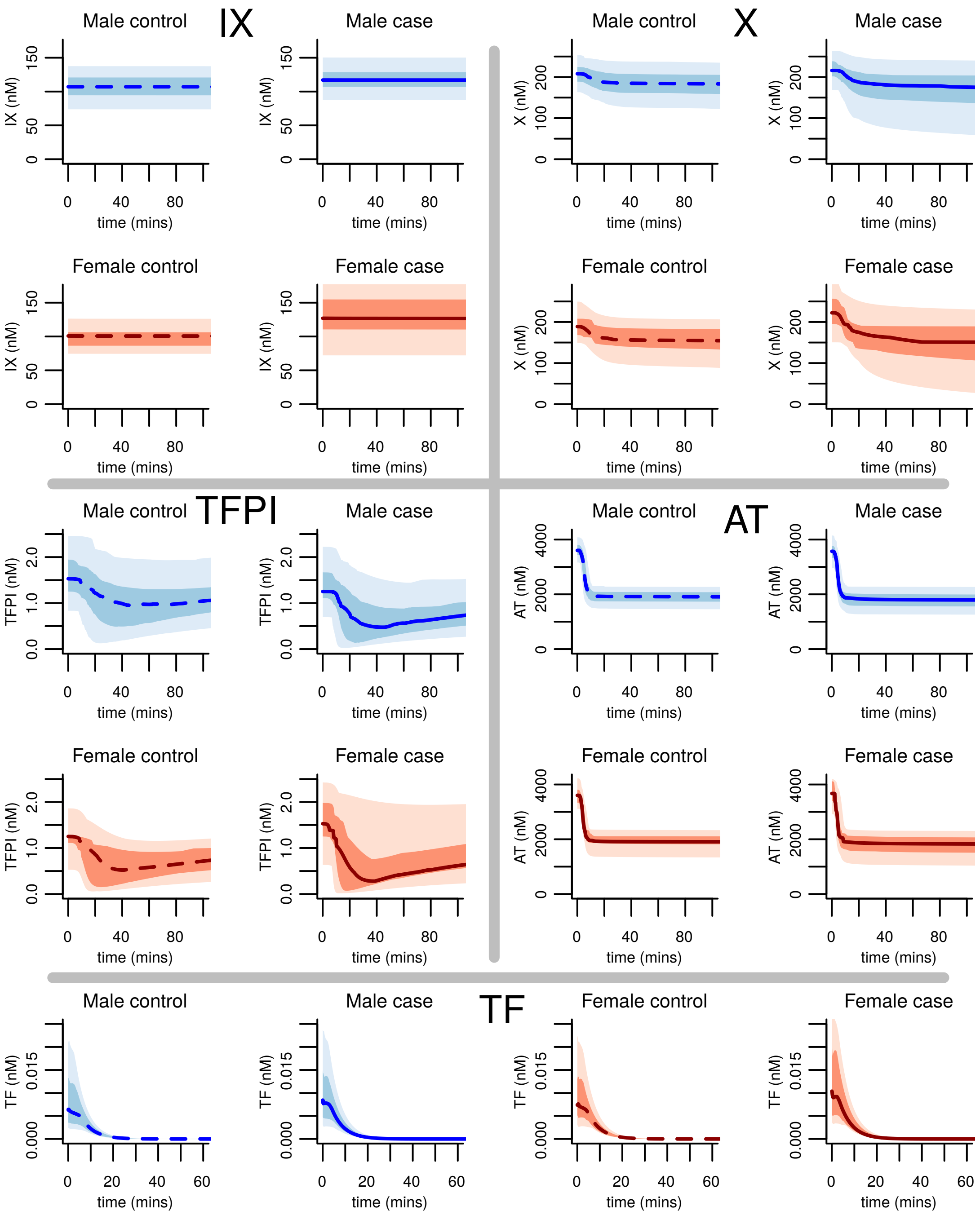

Figure SV: **Predictions for the depletion of procoagulants for males (blue) and females (red), both case (solid line) and control (broken line) populations (2 of 2).** See Figure SIV for further simulations.

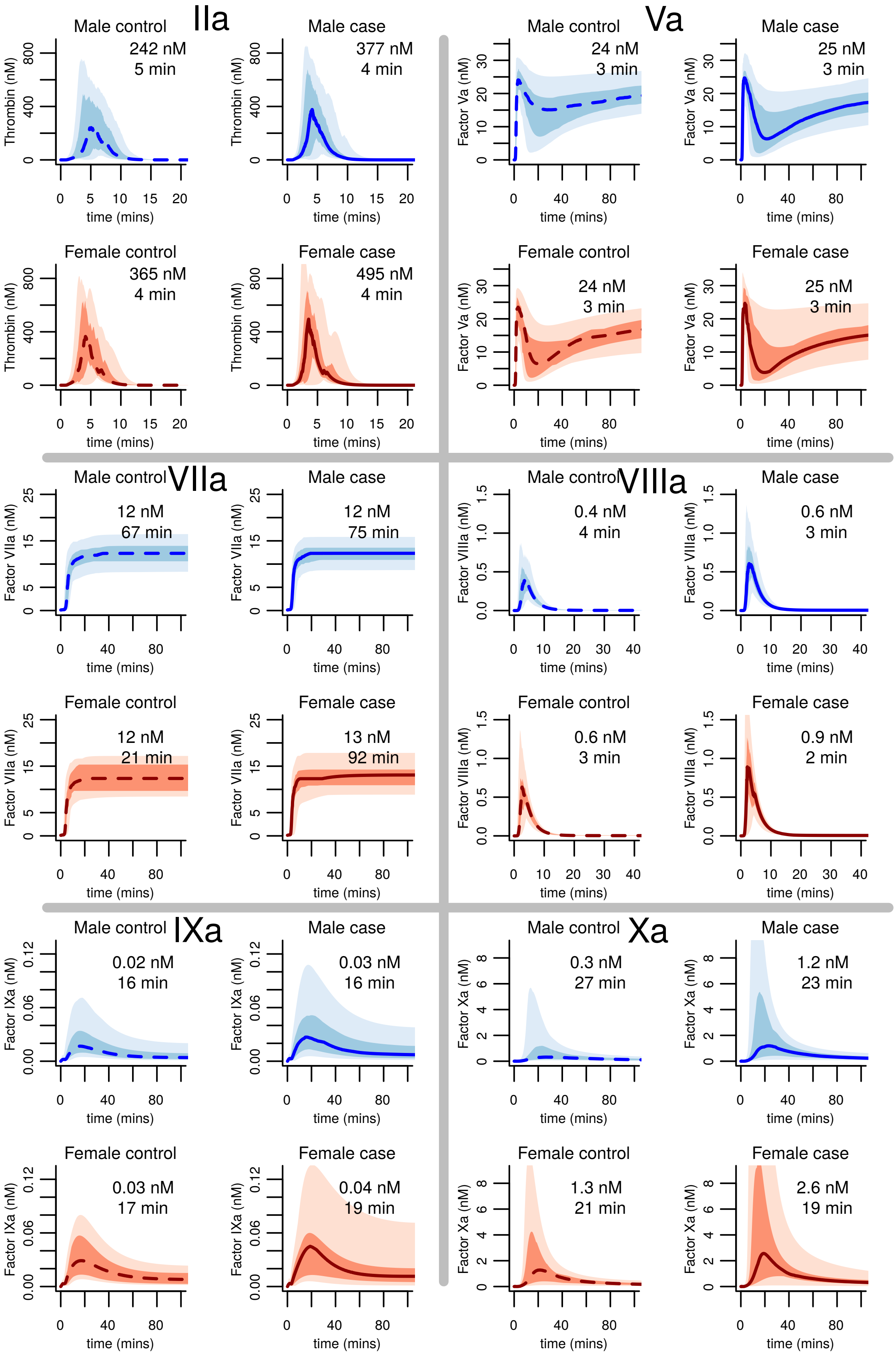

Figure SVI: **Predictions for the formation of active factors and complexes for males (blue) and females (red), both case (solid line) and control (broken line) populations of donors (1 of 2).** See Figure SVII for further simulations.

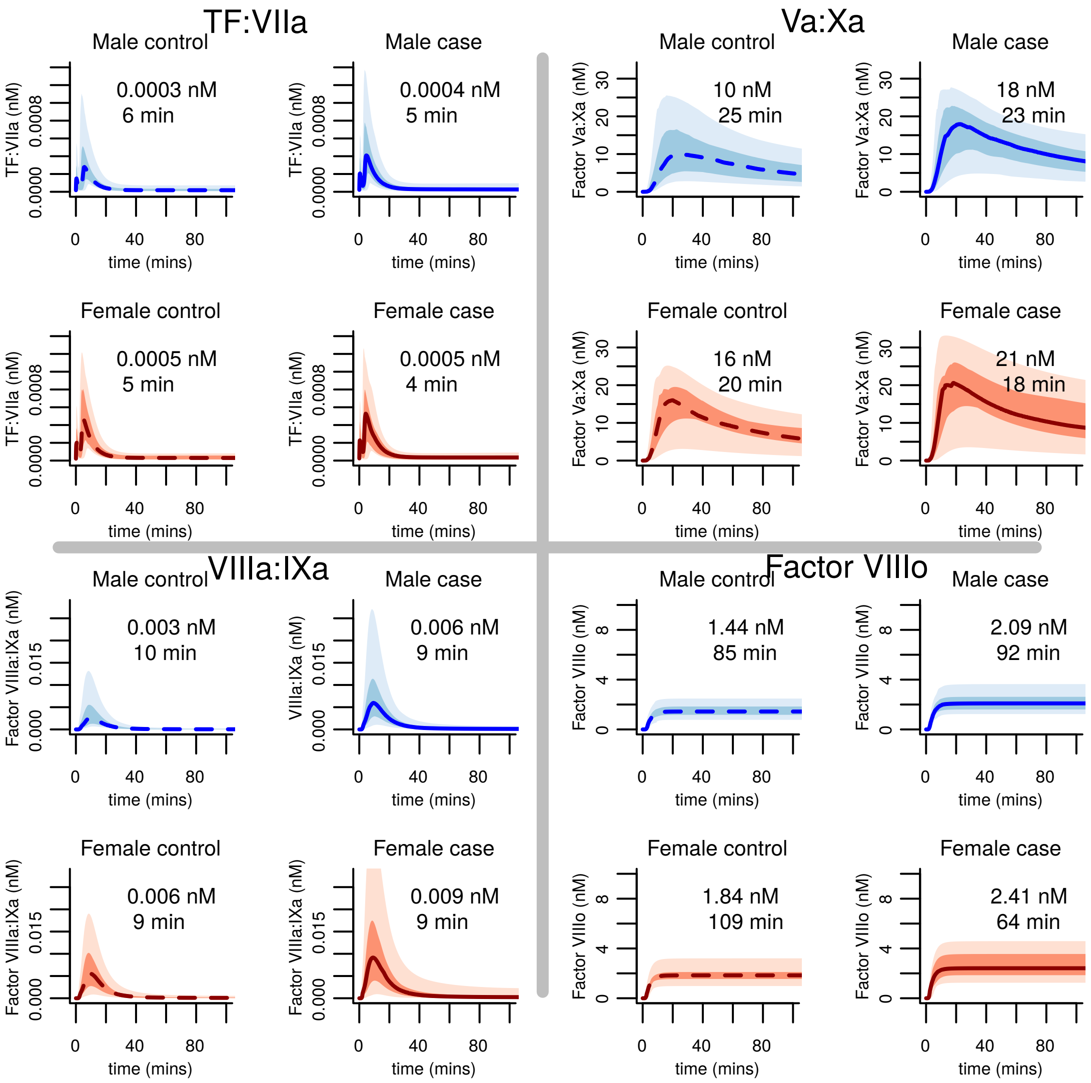

Figure SVII: **Predictions for the formation of active factors and complexes for males (blue) and females (red), both case (solid line) and control (broken line) populations of donors (2 of 2).** See Figure SVI for further simulations.

References

[1] Hockin MF, Jones KC, Everse SJ, Mann KG. (2002) A model for the stoichiometric regulation of blood coagulation. J. Biol. Chem., 277, 18322–18333.

[2] Butenas S, Orfeo T, Gissel MT, Brummel KE, Mann KG. The significance of circulating factor IXa in blood. J Biol Chem 2004;279(22):22875–22882. [PubMed: 15039440]

[3] Danforth, C.M., Orfeo, T., Mann, K.G., Brummel-Ziedins, K.E. and Everse, S.J., 2009. The impact of uncertainty in a blood coagulation model. Mathematical Medicine and Biology, 26(4), pp.323-336.

[4] Mitrophanov, A.Y. and Reifman, J., 2011. Kinetic modeling sheds light on the mode of action of recombinant factor VIIa on thrombin generation. Thrombosis research, 128(4), pp.381-390.

[5] Orfeo, T., Butenas, S., Brummel-Ziedins, K.E., Gissel, M. and Mann, K.G., 2010. Anticoagulation by factor Xa inhibitors. Journal of Thrombosis and Haemostasis, 8(8), pp.1745-1753.

[6] Brummel-Ziedins KE, Gissel M, Francis C, Queenan J, Mann KG. The effect of high circulating estradiol levels on thrombin generation during in vitro fertilization. Thromb Res 2009;124:505-7.

[7] Undas A, Gissel M, Kwasny-Krochin B, Gluszko P, Mann KG Brummel-Ziedins KE. (2010) Thrombin generation in rheumatoid arthritis: dependence on plasma factor composition. Thromb Haemost. 104(2):224-30.

[8] Gissel M, Undas A, Slowik A, Mann KG, Brummel-Ziedins KE. Plasma factor and inhibitor composition contributes to thrombin generation dynamics in patients with acute or previous cerebrovascular events. Thromb Res 2010;126:262-9.

[9] Mitrophanov, A.Y., Rosendaal, F.R. and Reifman, J., 2012. Computational analysis of intersubject variability and thrombin generation in dilutional coagulopathy. Transfusion, 52(11), pp.2475-2486.

[10] Orfeo T, Gissel M, Butenas S, Undas A, Brummel-Ziedins K.E. and Mann KG. (2011) Anticoagulants and the propagation phase of thrombin generation. PloS one. 6(11):e27852.

[11] Orfeo T, Butenas S, Brummel-Ziedins KE, Gissel M and Mann KG. (2010) Anticoagulation by factor Xa inhibitors. Journal of Thrombosis and Haemostasis. 8(8), pp. 1745-1753.

[12] Perzborn, E and Strassburger, J and Wilmen, A and Pohlmann, J and Roehrig, S and Schlemmer, K-H and Straub, A. (2005) In vitro and in vivo studies of the novel antithrombotic agent BAY 59-7939—an oral, direct Factor Xa inhibitor. Journal of Thrombosis and Haemostasis. 3(3), pp. 514-521.

[13] Hibbard, Lyndon S and Nesheim, Michael E and Mann, Kenneth G (1982) Progressive development of a thrombin inhibitor binding site. Biochemistry. 21(10), pp. 2285-2292
